# Regulatory mechanism of cysteine-dependent methionine biosynthesis in *Bifidobacterium longum*: insights into sulfur metabolism in gut microbiota

**DOI:** 10.1080/19490976.2024.2419565

**Published:** 2024-10-28

**Authors:** You-Tae Kim, Joon-Gi Kwon, Daniel J. O’Sullivan, Ju-Hoon Lee

**Affiliations:** a Department of Agricultural Biotechnology, Seoul National University, Seoul, Republic of Korea; b Department of Food and Animal Biotechnology, Seoul National University, Seoul, Republic of Korea; c Center for Food and Bioconvergence, Seoul National University, Seoul, Republic of Korea; d Research Institute of Agriculture and Life Sciences, Seoul National University, Seoul, Republic of Korea; e Department of Food Science and Nutrition, Center for Microbial and Plant Genomics, University of Minnesota, St. Paul, MN, USA

**Keywords:** *Bifidobacterium longum*, cysteine auxotroph, cysteine degradation, H_2_S production, methionine biosynthesis, sulfur utilization pathway

## Abstract

Sulfur, a critical element for bacterial growth, is not directly utilized by bifidobacteria, rendering the sulfur-containing amino acid biosynthesis pathway, particularly for cysteine and methionine, poorly understood. This research identifies six genes involved in this pathway through re-annotation of the *Bifidobacterium longum* DJO10A genome. These genes play crucial roles in bioconversion processes essential for cysteine utilization, highlighting its significance in sulfur metabolism. Our study uncovers a novel regulatory mechanism of these pathways under varying cysteine concentrations. We demonstrate a dual-pathway mechanism for methionine biosynthesis: one directly utilizing cysteine (trans-sulfurylation pathway) and another utilizing H_2_S derived from cysteine degradation (direct sulfurylation pathway). This regulatory dual-pathway mechanism is contingent on environmental cysteine levels, with both pathways activated at low cysteine levels, while higher levels predominantly engage the H_2_S-utilizing pathway. This investigation not only advances our understanding of DJO10A’s metabolic capabilities but also underscores the bacterium’s adaptability through sophisticated regulatory mechanisms for sulfur-containing amino acid biosynthesis. The elucidation of these pathways provides valuable insights into the survival strategies of bifidobacteria in the gut environment, where sulfur sources can vary greatly. Through detailed genomic, transcriptional, and enzymatic analyses, this study significantly contributes to the field of microbiology, offering a foundation for future research on gut microbiota metabolic pathways and their implications for host health.

## Introduction

The human large intestine plays a crucial role in reabsorbing water and various electrolytes from food waste to form feces.^1^ Additionally, it provides a complex microbial habitat where the gut microbiome interacts with the large intestine. The intestinal microbiota, together with the large intestine, play critical roles in health, exerting both beneficial and pathogenic effects via their interaction with the human host.^2^ However, the nutritional conditions in the large intestine are extremely harsh, limiting the ability of intestinal microbiota to survive, as only host-indigestible complex carbohydrates such as oligosaccharides, polyol, cellulose, hemicellulose, and other plant-derived dietary fibers are available.^3^ Therefore, intestinal microbes generally possess distinct metabolic pathways for digesting these complex carbohydrates. The intestinal microbial digestion of these non-digestible carbohydrates enhances the overall carbohydrate utilization efficiency from ingested foods.^4^

To understand these distinct metabolic pathways of gut microbes, the corresponding metabolic pathways were predicted and analyzed using complete genome sequences and bioinformatics. The analysis revealed that most gut microbes efficiently digest host-indigestible complex carbohydrates. However, these capabilities can vary depending on the gut environment and the available complex carbohydrates. For example, comparative genome analysis of *Bifidobacterium longum* subsp. *longum* DJO10A and *B. longum* subsp. *infantis* ATCC 15697 showed that the *B. longum* genome contains many genes for digesting plant-derived complex carbohydrates found in the adult diet, whereas the *B. infantis* genome contains specific genes for digesting human milk oligosaccharides (HMOs) from breastfeeding.^5,6^ Additionally, the *B. animalis* subsp. *lactis* genome has been found to contain genes only for the digestion of simple sugars, lacking genes for digesting plant-derived complex carbohydrates such as arabinofuran, xylan, and glycan, as *B. lactis* strains have adapted to specific growth conditions involving simple sugars such as those in commercial media.^5^ These results suggest that the different complex carbohydrate utilization preferences of bifidobacteria may be associated with strain adaptation to specific growth habitats. However, comparative genome analysis revealed no differences in the amino acid biosynthesis pathways from carbohydrate precursors despite different complex carbohydrate utilization preferences. Bifidobacteria digest different types of complex carbohydrates, but they all produce the same carbohydrate precursors via the *Bifidobacterium*-specific fructose-6-phosphate phosphoketolase (F6PPK) pathway and an impaired citric acid cycle from hexoses produced after digestion of complex carbohydrates.^5^ From these carbohydrate precursors, all 20 amino acids can be synthesized. However, the biosynthesis of sulfur-containing amino acids such as cysteine and methionine from these precursors remains not fully understood in the context of the human gut environment.

Sulfur-containing amino acids such as cysteine and methionine are crucial for various metabolic processes in bacteria, including protein synthesis, enzyme function, and antioxidative defense mechanisms.^7,8^ Without the biosynthesis of sulfur-containing amino acids, bacteria cannot synthesize certain essential proteins, leading to inhibited growth and potentially cell death in the given environment. Therefore, the biosynthesis of sulfur-containing amino acids is crucial for bacterial survival and cell proliferation. In addition, in *Mycobacterium tuberculosis*, methionine and cysteine are critical for maintaining redox homeostasis and synthesizing essential biomolecules.^9^ Cysteine is involved in the synthesis of mycothiol, a major antioxidant, while methionine contributes to the active methyl cycle, generating *S*-adenosylmethionine (SAM) for the biosynthesis of mycolic acids, menaquinone, and biotin. Disruption of methionine biosynthesis in *M. tuberculosis* leads to reduced SAM levels and significantly attenuates the bacterium’s virulence.^9^ In the gut environment, general gut bacteria have three different biosynthesis pathways for cysteine and methionine: (1) complete sulfur assimilation pathway (2) incomplete sulfur assimilation pathway without to adenosine 5’-phosphate (APS) kinase and phosphoadenosine-5’-phosphosulfate (PAPS) reductase (3) sulfite respiration pathway. The gut bacteria, *Escherichia coli* and *Salmonella enterica* utilize complete assimilatory sulfate reduction pathway,^10^ involving PAPS reductase, to obtain inorganic sulfur from the gut. Extracellular sulfate is transported into cells by the SulP (sulfate permease), CysP (sulfate/thiosulfate transport system substrate-binding protein), and CysZ (sulfate transport protein) transporters.^10^ Intracellular sulfate is converted to APS and then to PAPS, which is reduced to sulfite and further to sulfide by PAPS and sulfite reductases, respectively. Finally, cysteine is synthesized from *O-*acetyl serine and hydrogen sulfide, and is used for methionine synthesis.^11,12^ In addition, intestinal sulfate-reducing bacteria like *Desulfovibrio* and *Desulfobacter* use a unique dissimilatory sulfate reduction pathway, lacking APS kinase and PAPS reductase. Instead, APS reductase converts APS directly to sulfite, which is then used for cysteine and methionine synthesis.^13^ Few gut microbiota genera have a sulfite respiration pathway. *Bilophila wadsworthia* degrades taurine to sulfite, which is reduced to H_2_S for cysteine and methionine biosynthesis.^14^

However, bifidobacteria, one of the dominant genera in the gut, do not have ATP sulfurylase, APS kinase, PAPS reductase, and sulfite reductase in the genome, suggesting that they do not have sulfate assimilation pathway.^5^ Previous genome-level predictions of the amino acid biosynthesis pathway of *B. longum* DJO10A suggested direct assimilation of H_2_S produced by other intestinal bacteria, combined with *O-*succinyl homoserine by cystathionine γ-synthase (*metB*, BLD_0913), without a sulfate-reducing mechanism, producing cystathionine.^5^ This cystathionine is then converted to cysteine by cystathionine γ-lyase (BLD_1679). Additionally, cystathionine is converted to homocysteine by cystathionine-β-lyase (*metC*, BLD_0095) and subsequently to methionine by methionine synthase (*metE*, BLD_0538). However, a subsequent metabolic pathway study revealed that bifidobacteria are not autotrophic for cysteine and methionine but are instead auxotrophic for cysteine,^15^ likely due to the absence of H_2_S assimilation. Therefore, sulfur acquisition in the gut environment may be key to bifidobacteria survival, necessitating further investigation.^16^ In the human gut environment, organic and inorganic sulfur-containing compounds from ingested foods are supplemented and utilized by intestinal bacteria via their own sulfur assimilation pathways.^16^ However, clarifying the process of sulfide acquisition from the gut environment and the biosynthesis pathways of cysteine and methionine in bifidobacteria is still necessary to complete our understanding of amino acid biosynthesis in this genus. Enhanced sulfur amino acid metabolism in bifidobacteria can potentially improve the efficacy of probiotic formulations, promoting overall gut health and contributing to the prevention and treatment of gastrointestinal disorders. Insights into these metabolic pathways can inform the development of new therapeutic strategies targeting gut microbiota to enhance host health.

In the present study, our objective was to understand the mechanism of sulfur acquisition and the metabolic pathways of sulfur-containing amino acid biosynthesis in bifidobacteria. The enzyme genes were re-annotated using updated genome annotation databases, and their enzymatic activities were evaluated through heterologous gene expression. Furthermore, transcriptional analysis was performed to investigate how the expression of related enzyme genes (BLD_1130, BLD_0674, BLD_0538, BLD_0913, BLD_0095, and BLD_0914) is influenced by the presence of sulfur-containing amino acids in DJO10A. Based on these findings, the biosynthetic pathways for cysteine and methionine were elucidated. This metabolic characterization of sulfur-containing amino acids provides new insights into the amino acid biosynthesis processes in bifidobacteria and enhances our understanding of how bifidobacteria adapt and thrive in the human gut environment.

## Materials and methods

### *Genome analysis of* B. longum *DJO10A*

The sulfur acquisition and metabolic pathway-associated genes of *B. longum* DJO10A were re-annotated using the latest genome annotation databases. The BLASTP program was employed for amino acid sequence similarity searches to align the recent annotations of other *B. longum* strains.^17^ The InterProScan5 program was utilized for protein domain searches.^18^ The KEGG Automatic Annotation Server (KAAS) in conjunction with the KEGG database was applied to predict amino acid biosynthesis pathways and assign Enzyme Commission (EC) number to open reading frames (ORFs).^19^

Complete genome sequences from 20 *Bifidobacterium* species were obtained from GenBank, as detailed in Table S1. These genome sequences, along with their ORFs, were converted to generic feature format (gff3) files using Prokka version 1.14.^20^ These gff3 files facilitated pan-genome and comparative analyses of sulfur utilization pathway genes across the genomes, performed using the Roary program.^21^ For phylogenetic analysis, the average nucleotide identity (ANI) (using the BLAST algorithm ANIb with a 1,020-bp fragment length) was calculated with JSpecies,^22^ utilizing the FASTA format of all complete genome sequences.

### Bacterial strains, culture media, and materials

The bacterial strains and plasmids utilized in this study are listed in Table S2. *B. longum* subsp. *longum* DJO10A was cultivated at 37°C in MRS (Difco, USA) supplemented with 0.05% L-Cysteine·HCl (Sigma-Aldrich, USA) or in a chemically defined medium (CDM) based on M9 minimal medium, under anaerobic conditions. Anaerobic conditions were maintained using either the BBL anaerobic system (BD BBL GasPak, BD, USA) or serum vials with a gas mixture of N_2_, CO_2_, and H_2_ (80:10:10) in the headspace (Table S3). To supplement H_2_S in the medium, 2.5 g Na_2_S·9 H_2_O (Sigma-Aldrich) was dissolved in 100 ml of distilled water in a serum vial with 99.999% helium gas in the headspace as a stock solution. *Bacillus* (*Bac*.) *subtilis* was cultured at 37°C in tryptic soy (TS) media (Difco) and used as a positive control for the CDM medium. *E. coli* was cultured at 37°C in the Luria-Bertani (LB) medium (Difco) supplemented with ampicillin (50 μg/ml, final concentration) for gene cloning and expression hosting. Cell growth was measured by the optical density of the culture at an absorbance of 600 nm (OD_600_) using a DU730 spectrophotometer (Beckman Coulter, USA).

### RNA extraction and quantitative real time-PCR

After incubation, cells were harvested by centrifugation at maximum speed for 2 min at the mid-exponential growth phase (OD at 600 nm between 0.8 to 1.0). The RNA*later*^TM^ stabilization solution (Invitrogen, USA) was immediately added to the cell pellets. Total RNA was extracted using a PureLink RNA mini kit (Invitrogen) following the manufacturer’s instructions. RNA quality and concentration were measured using a NanoDrop 2000 device (Thermo Fisher, USA). Reverse transcription to cDNA was performed with a PrimeScript^TM^ RT reagent kit (Takara, Japan) under the following conditions: 15 min at 37°C and 5 sec at 85°C.

The mRNA expression levels were analyzed using TaqMan® technology via a quantitative real-time PCR (qRT-PCR) using the MG 2X qPCR master mix (MGMed, Korea) on a CFX96 system (Bio-Rad, USA). The qRT-PCR cycle was as follows: initial hold at 95°C for 3 min, followed by 39 cycles of 95°C for 10 sec and 55°C for 30 sec. Primers and probes were designed using the GeneScript primer design program (https://www.genscript.com/ssl-bin/app/primer), as listed in Table S4. The cycle threshold (*C*_*T*_) was automatically determined using the CFX Manager^TM^ software version 3.1 (Bio-Rad). All samples were analyzed in triplicate. Expression levels were normalized, and qRT-PCR data were analyzed using the 2^−ΔΔ*CT*^ method.^23^

### Genomic DNA extraction and PCR

Genomic DNA of *B. longum* DJO10A was extracted using a G-spin Genomic DNA extraction kit (Intron Biotechnology, Korea) and used as the template DNA for PCR. Target genes were amplified from the genomic DNA by PCR using specific primers containing restriction enzyme recognition sites (Table S4) and purified using an AxyPrep DNA gel extraction kit (Axygen Biosciences, USA). All PCR reactions were performed on a C1000 touch thermal cycler (Bio-Rad). The reaction mixtures (total volume of 30 μl) consisted of 1 μl of template, 1.5 μl of each primer (10 μM), 1.5 μl of MgSO_4_ (25 mM, Toyobo, Japan), 3 μl of deoxynucleoside triphosphates (2 mM each, Toyobo), and 1 μl of KOD-Plus- high-fidelity DNA polymerase (1 U/μl, Toyobo). PCR conditions were as follows: one cycle of 94°C for 3 min; 27 cycles of 94°C for  0.5 min, 57°C for  0.5 min, and 68°C for 1.5 min; and a final extension at 68°C for 3 min. PCR products were purified using PCR clean-up S & V kits (Bionics, Korea).

### Gene cloning and heterologous gene expression

Purified PCR products were digested with restriction enzymes (New England BioLabs, Inc., USA) following standard laboratory procedure.^24^ Digested PCR products were ligated into pET15b vectors linearized by double digestion with *Nhe*I-*Xho*I or *Nde*I-*Bam*HI, respectively, using T4 DNA ligase (Promega, USA) at 16°C for 4 h. Ligated plasmids were transformed into *E. coli* DH5α. Gene-harboring plasmids were then transformed into *E. coli* BL21 (DE3) by heat shock at 42°C for 90 sec for heterologous gene expression. Recombinant cells were grown in a LB medium supplemented with 50 μg/ml ampicillin at 37°C until the OD at 600 nm was 0.5. Overexpression of recombinant proteins was induced by adding 0.5 mM of isopropyl-β-D-thiogalactopyranoside (IPTG; final concentration) and cultivating at 18°C for 24 h.

### Purification of recombinant enzymes

Cells were harvested by centrifugation at 7,000 × g at 4°C for 10 min. Each cell pellet was resuspended in 10 ml of lysis buffer containing 50 mM NaH_2_PO_4_, 300 mM NaCl, 10 mM imidazole, and 0.1 mM pyridoxal 5’-phosphate (PLP). The cells were disrupted by an EpiShear^TM^ probe sonicator (10 sec/10 sec pulse for 20 min, 45% amplitude; Active Motif, USA) in an ice bath. Crude protein was obtained by centrifugation (7,000 × *g*, 4°C for 20 min) and purified from the supernatant using Ni-NTA affinity column chromatography (Qiagen, USA). Purified enzymes were collected using an elution buffer (lysis buffer modified with 500 mM imidazole). Imidazole was removed by exchanging the enzyme buffer with 20 mM sodium citrate buffer pH 6.0 using an Amicon Ultra-15 device (Millipore, USA). Protein concentration was estimated using a Pierce BCA protein assay kit (Thermo Fisher), and molecular size was estimated by 10% SDS-PAGE.

### Optimum pH assay

Enzyme reactions were performed at 37°C with 200 μl of a 50 mM enzyme buffer, 0.1 mM of PLP, 10 mM substrate (homocysteine, serine, *O*-succinyl-homoserine, *O*-acetyl-homoserine, *O*-acetyl-serine, cystathionine, cysteine, and H_2_S) and 0.1 mg recombinant enzyme. For the optimum pH assay, 50 mM enzyme buffer with sodium citrate (pH 4.0, 5.0, and 6.0) and sodium phosphate buffer (pH 6.0 and 7.0) was used.

### LC-MS/MS spectrometry

Seven standard substrates (homoserine, serine, *O*-succinyl homoserine, *O*-acetyl homoserine, cystathionine, homocysteine, and cysteine) were analyzed in positive ion mode to produce MS/MS spectra via an ESI (electrospray ionization) source using liquid chromatography – mass spectrometry (6410B, Rapid Resolution Liquid Chromatography system; Agilent, USA). The mobile phase solvent was a mixture of 95% methanol-5% acetonitrile with a flow rate of 0.15 ml/min using binary pump. Parameters were as follows: scan type, multiple reaction monitoring (MRM); fragmentor voltage, 135 V; nebulizer pressure, 30 psi; gas temperature, 350°C; gas flow rate, 6 l/min; injection volume of the low-flow sampler, 5 μl; diode array detector (DAD) signal wavelength, 254 nm; chromatogram type, total ion chromatogram (TIC). The observed mass-to-charge scan width ranged from 25 to 250 m/z. Enzyme reaction products were identified based on the specific ion production from each sample.

### Quantification assays

Colorimetric assays quantified the enzyme reaction products of cysteine, H_2_S, acetate, and succinate. Cysteine quantification followed Rocchiccioli’s method.^25^ Cysteine concentration was determined using a standard curve (10 to 200 μM). In addition, H_2_S in the solution was quantified using Siegel’s protocol with *N,N*-dimethyl-*p*-phenylenediamine (DMPD), with modifications to the reaction volume.^26^ A solution containing H_2_S was mixed with equal volumes of 30 mm FeCl_3_ in 1.2 M HCl and 20 mM DMPD in 7.2 M HCl, incubated for 30 min at room temperature, and absorbance measured at 670 nm (Beckman Coulter). H_2_S concentration was determined using a standard curve (10 to 200 μM). Released acetate, succinate, and pyruvate were quantified using respective colorimetric assay kits: Acetate colorimetric assay kit, Succinate colorimetric assay kit, and Pyruvate assay kit (all from Sigma-Aldrich).

### Kinetic measurements

All kinetic measurements were performed with a reaction volume of 30 μl containing 50 mM of a sodium phosphate buffer (pH 7.0), 0.1 mM of PLP, and 27.3 U/mg of each recombinant enzyme at 37°C. The reaction was initiated by the addition of each enzyme and terminated by adding 30 μl of phenol:chloroform:isoamyl alcohol (25:24:1, Thermo Scientific). Kinetic parameters (*K*_m_) for the reactions were determined using appropriate colorimetric assay kits (Sigma-Aldrich) and cysteine quantification with Rocchiccioli’s method.^25^ All concentrations were measured in triplicate, and data were calculated according to the Hanes-Woolf equation.^27^

### Analysis of enzyme sequence similarity network and enzyme abundance in metagenome datasets

The Sequence Similarity Network (SSN) analysis for enzyme sequences BLD_0674 and BLD_0913 was conducted using the Enzyme Function Initiative-Enzyme Similarity Tool (EFI-EST) based on the UniProt database, with a query e-value of 10^−5^. Clusters were distinguished using an alignment score threshold of 225. Detailed exploration and visualization of the SSN were performed using Cytoscape software. To analyze the abundance of these enzymes (BLD_0674 and BLD_0913) in the human body, a total of 380 metagenomic shotgun sequencing datasets from the Human Microbiome Project (HMP) were used. Profiling of the clusters of interest was performed using the ShortBRED program within the EFI-CGFP platform.^28,29^

## Results

### *Prediction of the sulfur utilization pathway in* B. longum *DJO10A*

**Figure 1.**
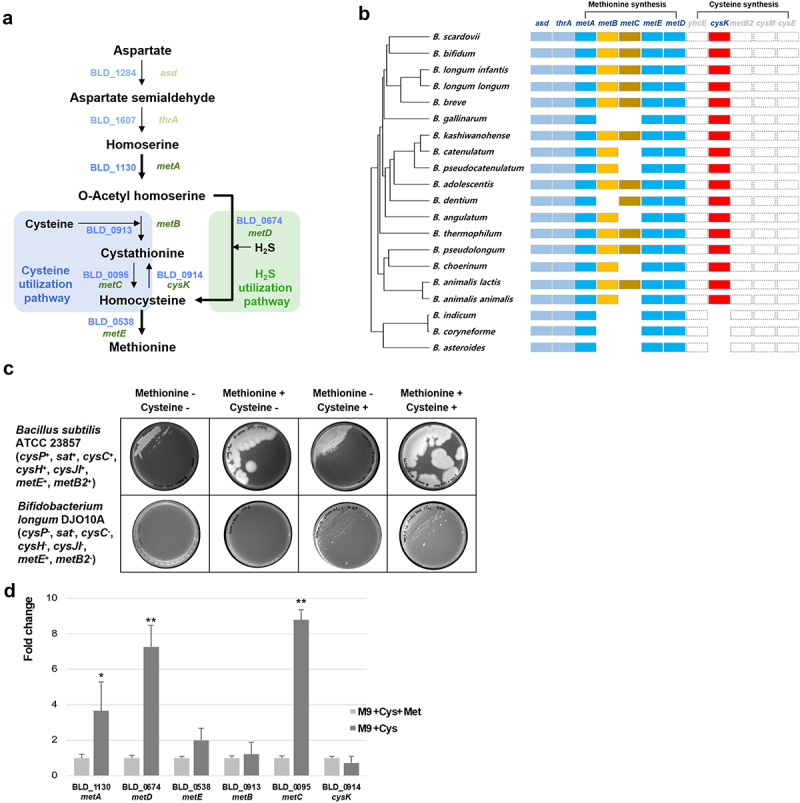
Cysteine and methionine metabolism of *B. longum* DJO10A. (a) Predicted sulfur utilization pathway of *B. longum* DJO10A. Aspartate generated as a byproduct of the citric acid cycle (oxaloacetate) is reduced to aspartate semialdehyde by aspartokinase (BLD_1284). Aspartate semialdehyde is then reduced to homoserine by homoserine dehydrogenase (BLD_1607). Homoserine is converted to *O*-acetyl homoserine by BLD_1130, homoserine *O*-acetyltransferase. However, *O*-acetyl homoserine is converted to two different products, cystathionine with cysteine and homocysteine with H_2_S, designated as the trans- and direct sulfurylation pathway, respectively. In the trans-sulfurylation pathway, cystathionine γ-synthase (BLD_0913) detaches the acetate residue from *O*-acetyl homoserine and attaches cysteine from the environment, generating cystathionine. The next step is conversion to homocysteine by cystathionine β-lyase (BLD_0095), with the homocysteine converted again to methionine by methionine synthase (BLD_0538). In the direct sulfurylation pathway, *O-*acetylhomoserine sulfhydrylase (BLD_0674) detaches the acetate residue from *O*-acetyl homoserine and attaches H_2_S, generating homocysteine. This is subsequently also converted to methionine by methionine synthase (BLD_0538). (b) Pan-genome analysis of the metabolic pathway with 20 bifidobacteria (Table S1). Solid squares are conserved proteins (over 70% protein sequence identity), blanks are non-conserved proteins, and dashed squares are missing proteins in the genus of *Bifidobacterium*. (c) Auxotrophy test on the chemically defined media (CDM). Cysteine or methionine auxotrophy test of *B. longum* DJO10A with *Bac. subtilis* ATCC 23857 as a positive control at different combinations of cysteine and/or methionine. (d) Transcriptional analysis of sulfur utilization pathway-associated genes in *B. longum* DJO10A. Relative fold changes of mRNA expression levels were compared between the conditions of CDM+Cys+Met and CDM+Cys. Statistical differences were examined by Student’s t-test. *p < 0.05, **p < 0.01. *asd*, aspartate-semialdehyde dehydrogenase; *thrA*, homoserine dehydrogenase; *metA*, homoserine *O*-acetyltransferase; *metB*, cystathionine γ-synthase; *metC*, cystathionine β-lyase, *metE*, methionine synthase; *cysD*, *O*-acetylhomoserine sulfhydrylase; *cysK*, cysteine synthase; *mccB*, cystathionine γ-lyase; *cysP*, sulfate/thiosulfate transport system substrate-binding protein; *sat*, sulfate adenylyltransferase; *cysC*, adenylylsulfate kinase; *cysH*, phosphoadenosine phosphosulfate reductase; *cysJI*, sulfite reductase; Cys, Cysteine; Met, methionine.

The complete genome sequence of *B. longum* DJO10A was published in 2008, but the annotation information still needs to be corrected for the exact metabolic pathway.^30^ The genes associated with the sulfur utilization pathway were selected, and their newly updated annotations and functions are listed in Table S5. To clarify the metabolism in bifidobacteria, the previous sulfur utilization pathway was updated with the re-annotated functions (Figure 1(a)). This pathway of *B. longum* DJO10A was re-predicted with Gram-positive bacteria containing a sulfur utilization pathway.^15, 31–33^ This pathway consists of the H_2_S utilization pathway (direct sulfurylation), where sulfur is obtained from H_2_S to convert *O*-acetyl homoserine to homocysteine, and the cysteine utilization pathway (trans-sulfurylation), where sulfur is obtained from cysteine and convert to homocysteine via cystathionine. Previously, it was reported that *Corynebacterium glutamicum* has a similar sulfur utilization pathway consisting of trans- and direct sulfurylation, supporting this prediction.^34^ However, the predicted sulfur utilization pathway still needs to be validated experimentally and compared with the sulfur utilization pathways of other bifidobacteria. Additionally, it is necessary to clarify the selection of these trans- or direct sulfurylation pathways for methionine biosynthesis.

### *Comparative analysis of the sulfur utilization pathway in other* Bifidobacterium *species*

As of March 2024, 244 complete genome sequences of the genus *Bifidobacterium* are available in the GenBank database. Among them, 20 representative complete genome sequences from each species were selected for a comparative analysis of the sulfur utilization pathways (Table S1). From the pan-genome analysis, five shared genes (*asd*, aspartate-semialdehyde dehydrogenase; *thrA*, homoserine dehydrogenase; *metA*, homoserine *O*-acetyltransferase; *metE*, methionine synthase; *cysD*, *O*-acetyl homoserine sulfhydrylase) with over 70% of homologies along with three partially shared genes (*metB*, cystathionine γ-synthase; *metC*, cystathionine β-lyase; *cysK*, cysteine synthase) were detected (Figure 1(b)). These eight genes were predicted to synthesize methionine from aspartate (Figure 1(a)). Among them, ten species of bifidobacteria have a complete sulfur utilization pathway identical to that of *B. longum* DJO10A, suggesting that this predicted sulfur utilization pathway for methionine biosynthesis is common in bifidobacteria. However, three partially shared genes of the trans-sulfurylation pathway are not completely present in the other ten bifidobacterial genomes, suggesting that these bifidobacterial species may only use the direct sulfurylation pathway and not the trans-sulfurylation pathway (Figure 1(a)). Interestingly, most of these species typically originate from the feces of animals and insects, implying that the incomplete trans-sulfurylation pathway may be due to the origins of these bifidobacterial species (Table S1). This comparative analysis indicates that bifidobacteria may share a distinct pathway for methionine biosynthesis from aspartate. However, cysteine from this sulfur utilization pathway is not generated but instead utilized for methionine biosynthesis, implying that bifidobacteria may be cysteine-dependent.

### Cysteine requirement for the growth of bifidobacteria

The sulfur assimilation mechanism is well known in other intestinal bacteria, including *E. coli, S*. Typhimurium, and *Bac. subtilis*.^10,11^ Compared to these bacteria, bifidobacteria lack a sulfate-reducing pathway to produce sulfide and no cystathionine γ-lyase to produce cysteine, supporting the cysteine-dependent characteristic of bifidobacteria.^5^ To evaluate this cysteine-dependent feature of bifidobacteria, *B. longum* was cultivated in chemically defined media (CDM) supplemented with sulfate-(MgSO_4_) or sulfur-containing amino acids (cysteine or methionine) (Table S3 and Figure 1(c)). *Bac. subtilis*, used as a positive control, grew fully in this medium with any supplementary sulfur sources, indicating it has sulfate-reducing activity and can produce cysteine and methionine (Figure 1(c)). Its complete genome sequence contains all related genes for these activities (*cysP*, sulfate/thiosulfate transport system substrate-binding protein; *sat*, sulfate adenylyltransferase; *cysC*, adenylyl-sulfate kinase; *cysH*, phosphoadenosine phosphosulfate reductase; *cysJI*, sulfite reductase; *metE*, methionine synthase; *mccB*, cystathionine γ-lyase), supporting this result.^11^ However, *B. longum* DJO10A grew only with cysteine, suggesting that this strain requires cysteine for growth (Figure 1(c)). This result is consistent with the cysteine-dependent feature of the sulfur utilization pathway in bifidobacteria. While previous work reported the prediction of a symporter protein and transport system for cysteine intake in *B. bifidum* PRL2010,^15^ *B. longum* DJO10A possesses only an ABC-type amino acid transport system (BLD_0020 to BLD_0023), not a symporter system, supporting this cysteine-dependent feature. Interestingly, methionine supplementation was not necessary for the growth of bifidobacteria, likely because the sulfur utilization pathway can generate methionine, suggesting that bifidobacteria may not be methionine-dependent (Figure 1(c)). Therefore, this result substantiates that *B. longum* DJO10A is a cysteine-auxotroph.

### Transcriptional analysis of cysteine utilization genes in a methionine-starvation condition

To verify the cysteine-dependent feature of *B. longum* DJO10A, the mRNA expression patterns of cysteine utilization genes were compared with or without methionine supplementation in a CDM medium containing cysteine. In the absence of methionine supplementation, the mRNA expression levels of BLD_1130 (*metA*; homoserine *O*-acetyltransferase), BLD_0674 (*metD*; *O-*acetylhomoserine sulfhydrylase), BLD_0538 (*metE*; methionine synthase), BLD_0913 (*metB*; cystathionine γ-synthase), BLD_0095 (*metC*; cystathionine β-lyase), and BLD_0914 (*cysK*; cystathionine β-synthase) were quantified using qRT-PCR (Figure 1(d)). Five genes, except for BLD_0914, were relatively highly expressed in the methionine-deficient condition compared to the methionine-supplemented condition. This suggest the involvement of both trans- and a direct sulfurylation pathway in methionine biosynthesis. The activation of the trans-sulfurylation pathway indicates that cysteine in the medium was used and converted to methionine via this pathway under methionine-starvation condition (Figure 1(a)). However, although the direct sulfurylation pathway is activated for methionine biosynthesis, the origin of H_2_S in the medium is not yet understood. Notably, BLD_1130, BLD_0674, and BLD_0095 showed significant differential regulation (*p* < 0.05), suggesting that *B. longum* DJO10A expresses the mRNAs of these genes to produce methionine under a methionine-starvation condition. This result clarifies that cysteine is used and converted to methionine through the trans-sulfurylation pathway to compensate for methionine starvation. Interestingly, the mRNA expression of BLD_0914 was slightly reduced, suggesting that the expression of this gene may be repressed for methionine biosynthesis (Figure 1(d)). The associated enzyme activities need to be validated experimentally to confirm this mRNA expression pattern for the bioconversion of cysteine to methionine in the pathway. Additionally, further experimental confirmation of the H_2_S origin is required to understand the activation of the direct sulfurylation pathway under a methionine-starvation condition.

### Roles of key enzymes in methionine biosynthesis using cysteine or H_2_S

**Figure 2.**
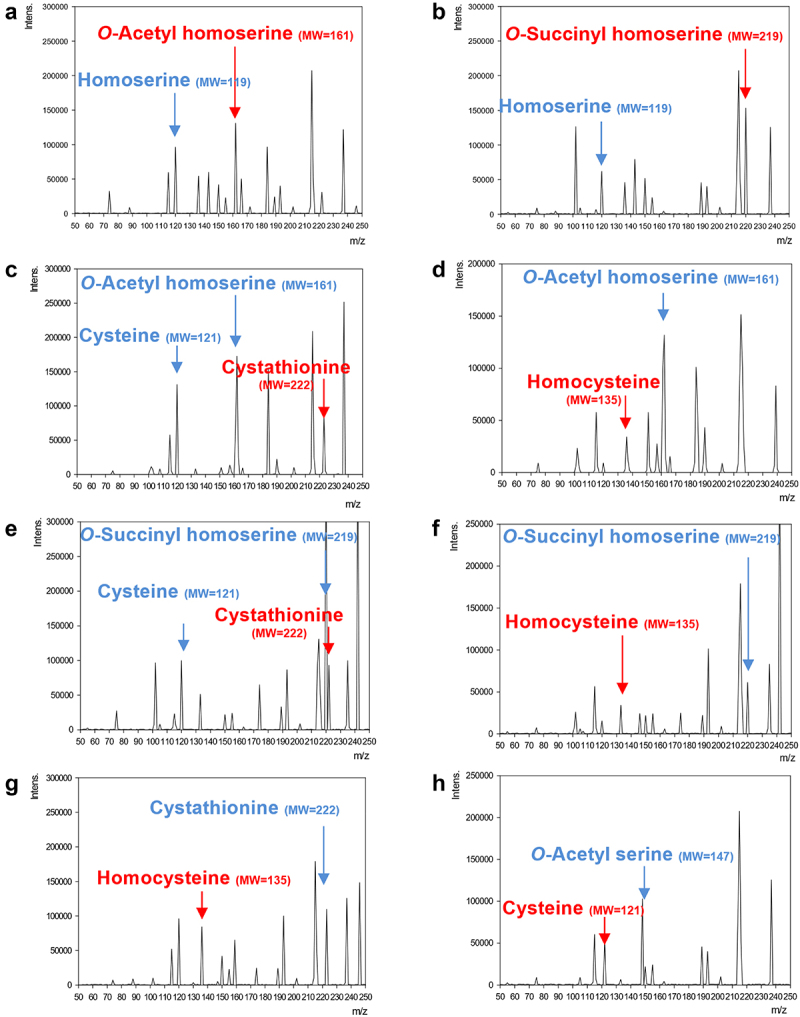
Electrospray ionization mass spectrum assay with recombinant enzymes from *B. longum* DJO10A. Illustrated are the mass spectra of enzymatic reaction with BLD_1130 (a and b), BLD_0674 (c and d), BLD_0913 (e and f), BLD_0095 (g), and BLD_0914 (h). Substrates (blue characters and arrows) and products (red characters and arrows) of the enzyme reaction are listed in Table S6.

To clarify the functions of the key enzymes for methionine biosynthesis, BLD_1130, BLD_0674, BLD_0913, BLD_0095, and BLD_0914 were cloned, expressed, and purified using the *E. coli* gene expression with the Ni-NTA purification (Figure S1). Subsequently, the optimum pH for an enzymatic reaction was determined to pH 7.0 using 50 mM of a sodium phosphate buffer. The enzymatic reaction of substrates with purified recombinant and the detection of targeted chemicals were identified using LC-MS/MS analysis (Figure 2 and Table S6).

The enzymatic reaction between homoserine *O*-acetyltransferase (BLD_1130) and acetyl-CoA or succinyl-CoA shows that this enzyme has dual enzymatic functions to transfer acetyl or succinyl residue to homoserine, resulting in *O*-acetyl homoserine or *O*-succinyl homoserine (Figures 2a,b). This suggests that homoserine can be converted to *O*-acetyl homoserine or *O*-succinyl homoserine by this enzyme. However, the predicted sulfur utilization pathway only involves the bioconversion of homoserine to *O*-acetyl homoserine (Figure 1(a)). Therefore, this predicted sulfur utilization pathway must be updated with the new bioconversion pathway of homoserine and succinyl-CoA to *O*-succinyl homoserine.

Based on the predicted trans-sulfurylation pathway, *O*-acetyl homoserine with cysteine was converted to cystathionine by cystathionine γ-synthase (BLD_0913) (Figure 1(a)). However, this enzymatic reaction did not produce cystathionine (Table S6). Instead, *O*-acetyl homoserine sulfhydrylase (BLD_0674) with cysteine produced cystathionine (Figure 2(c)). Previously, the predicted direct sulfurylation pathway showed that BLD_0674 uses H_2_S for homocysteine production (Figure 1(a)). To validate this activity, the enzymatic reaction with H_2_S produced homocysteine, confirming this function (Figure 2(d)). This result indicates that BLD_0674 also has dual enzymatic activities: cystathionine production with cysteine and homocysteine production with H_2_S.

To elucidate the enzyme activity of cystathionine γ-synthase (BLD_0913), the enzymatic reaction with cysteine or H_2_S was conducted, resulting in its dual functions for the bioconversion of *O*-succinyl homoserine to cystathionine or homocysteine (Figures 2e,f, respectively). It was previously suggested that BLD_0913 may be responsible for the bioconversion of *O*-acetyl homoserine with cysteine to cystathionine in the predicted sulfur utilization pathway (Figure 1(a)), which is different from this enzymatic characterization result of BLD_0913. The subsequent enzyme characterizations of BLD_0674 and BLD_0913 substantiate that BLD_0674 with cysteine is responsible for this bioconversion of *O*-acetyl homoserine to cystathionine, and BLD_0913 with cysteine or H_2_S is responsible for the bioconversion of *O*-succinyl homoserine to cystathionine or homocysteine, respectively. Therefore, the enzymatic reactions for the productions of cystathionine or homocysteine from *O*-succinyl homoserine with cysteine or H_2_S should be updated to the predicted pathway.

Previously, the predicted trans-sulfurylation pathway showed that cystathionine β-lyase (BLD_0095) converts cystathionine to homocysteine. To validate this enzyme reaction, cystathionine was treated with this enzyme and produced homocysteine, substantiating the predicted enzyme function (Figure 2(g)).

The predicted trans-sulfurylation pathway showed that cystathionine β-synthase (BLD_0914) converts homocysteine to cystathionine as a reverse reaction (Figure 1(a)). To verify this enzymatic function, acetyl serine as a substrate was treated with this enzyme with H_2_S and produced cysteine, suggesting that *B. longum* also synthesizes cysteine from acetyl serine and H_2_S (Figure 2(h)). However, transcriptional analysis of this enzyme revealed that the corresponding gene expression was very low and even repressed under a methionine-starvation condition (Figure 1(d)), suggesting that this enzyme may be not fully functional for cysteine biosynthesis in bifidobacteria. The exact function of this enzyme in bifidobacteria remains to be elucidated, most likely in other metabolic pathways.

### H_2_S production from cysteine and utilization for homocysteine biosynthesis

As previously mentioned, the origin of H_2_S in bifidobacteria is not clearly understood. However, the acquisition of H_2_S may be important for the bioconversion of *O*-acetyl homoserine to homocysteine in the predicted direct sulfurylation pathway (Figure 1(a)). In addition, H_2_S is used in the enzymatic reactions with *O*-acetyl homoserine sulfhydrylase (BLD_0674) and cystathionine γ-synthase (BLD_0913) (Figures 2d,f). Previous studies reported that various types of intestinal bacteria, such as *Pseudomonas aeruginosa*, *Staphylococcus aureus*, and *E. coli*, can degrade cysteine to H_2_S using cystathionine β-synthase, cystathionine γ-lyase, or 3-mercaptopyruvate sulfurtransferase, respectively.^35^ However, characterization of cystathionine β-synthase (BLD_0914) in bifidobacteria revealed that it synthesizes cysteine from acetyl serine and H_2_S and does not degrade cysteine to H_2_S; moreover, this enzyme may not even be functional in bifidobacteria (Figures 1d and 2h). Additionally, cystathionine γ-lyase and 3-mercaptopyruvate sulfurtransferase are not present in the genome of *B. longum* DJO10A. Therefore, it is necessary to determine which gene is responsible for cysteine degradation for H_2_S production in bifidobacteria.

**Figure 3.**
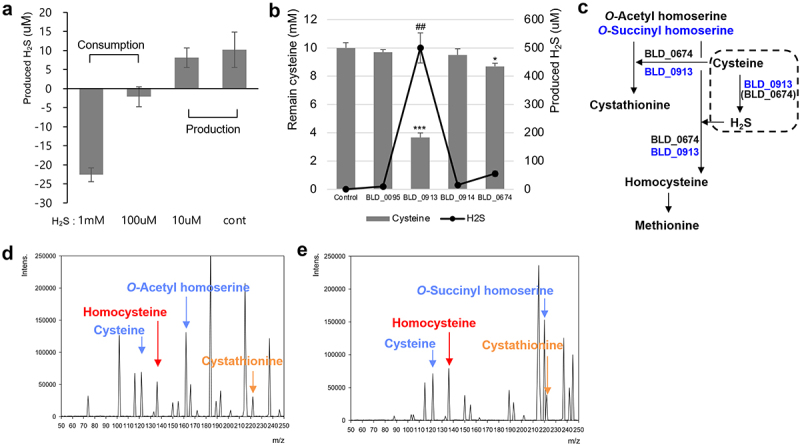
Determination and characterization of H_2_S production-associated enzymes in *B. longum* DJO10A. (a) H_2_S consumption or production in DJO10A according to H_2_S concentration. H_2_S concentration was determined by measuring the difference between its initial levels and the levels after incubation with/without H_2_S under conditions of 0.03% cysteine. (b) The amounts of produced H_2_S and reduced cysteine. (c) Newly updated additional pathway in sulfur utilization pathway. (d) Enzymatic bioconversion of *O-*acetyl homoserine/cysteine to homocysteine and cystathionine, (e) *O-*succinyl homoserine/cysteine to homocysteine and cystathionine by purified enzyme mixture from BLD_0674 and BLD_0913. Blue characters and arrows are substrates, and reds and yellows are products.

To verify H_2_S production by *B. longum* DJO10A, this strain was cultivated with cysteine and different concentrations of H_2_S supplementation (Figure 3(a)). Interestingly, DJO10A consumed H_2_S at a high concentration (0.1 to 1 mM) but produced it at low concentration (0 to 0.01 mM), indicating that *B. longum* DJO10A can indeed produce H_2_S when the amount of H_2_S is insufficient. Therefore, it is necessary to elucidate which enzyme in the sulfur utilization pathway is involved in H_2_S production from cysteine. To validate this, four enzymes, cystathionine β-lyase (BLD_0095), cystathionine γ-synthase (BLD_0913), cystathionine β-synthase (BLD_0914), and *O*-acetyl homoserine sulfhydrylase (BLD_0674), were selected and tested for cysteine degradation and H_2_S production. Strikingly, cystathionine γ-synthase (BLD_0913) degraded more than 6 mM of cysteine, leaving 500 μM of H_2_S in the media, and *O*-acetyl homoserine sulfhydrylase (BLD_0674) used 1 mM of cysteine, with 50 μM of H_2_S detected after enzymatic reactions (Figure 3(b)). Based on this result, cystathionine γ-synthase (BLD_0913) may play a major role in the production of H_2_S from cysteine, and the produced H_2_S may be used for the bioconversion of *O*-acetyl homoserine or *O*-succinyl homoserine to homocysteine by BLD_0674 or BLD_0913, respectively (Figure 3(c)). Interestingly, these enzymes also have additional functions related to the bioconversion of *O*-acetyl homoserine and *O*-succinyl homoserine to cystathionine by direct addition of cysteine. Cystathionine, in this case, is further converted to homocysteine. Consequently, cystathionine γ-synthase (BLD_0913) is responsible for cysteine degradation and H_2_S production, substantiating that H_2_S is produced from cysteine degradation in bifidobacteria.

As previously suggested, cystathionine γ-synthase (BLD_0913) produce H_2_S from added cysteine, with the produced H_2_S then used for the bioconversion to homocysteine or cystathionine by *O*-acetyl homoserine sulfhydrylase (BLD_0674) and cystathionine γ-synthase (BLD_0913). To experimentally validate this suggested pathway of cysteine degradation and H_2_S production, reactions with a mixture of cysteine and these two enzymes were performed with different substrates, *O*-acetyl homoserine (Figure 3(d)) or *O*-succinyl homoserine, without addition of cysteine (Figure 3(e)). As expected, *O*-acetyl homoserine and *O*-succinyl homoserine were converted to homocysteine using the H_2_S from cysteine degradation or were converted with cysteine to cystathionine. Therefore, all remaining substrates (cysteine; *O*-acetyl homoserine or *O*-succinyl homoserine) and all products (homocysteine; cystathionine) were detected (Figures 3d,e), substantiating this.

### Preference of a specific pathway in the sulfur utilization pathway

**Figure 4.**
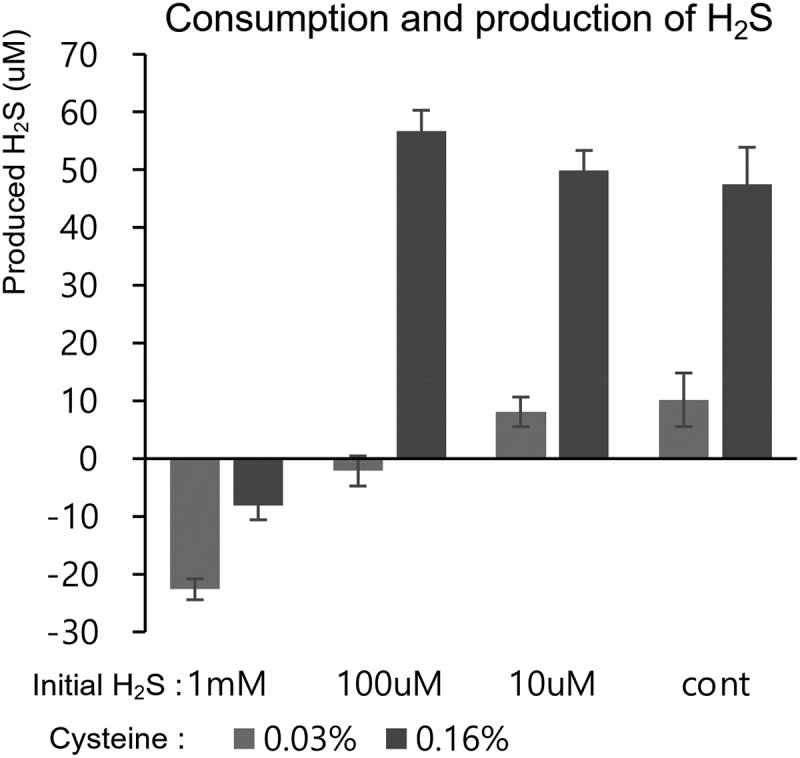
Comparative quantification of H_2_S production in *B. longum* DJO10A, according to different concentration combinations of H_2_S (0, 0.01, 0.1, and 1 mM) and cysteine (0.03 and 0.16%).

Previously, it was believed that the regulation of H_2_S consumption and production in *B. longum* DJO10A was solely dependent on the concentration of H_2_S in the medium (Figure 3(a)). In this result, H_2_S produced in the medium is derived from the degradation of cysteine. Consequently, it is essential to evaluate the relationship between H_2_S consumption and production with respect to the concentrations of both hydrogen sulfide and cysteine. Consistent with previous findings, a high concentration of H_2_S (1 mM) combined with a low concentration of cysteine (0.03%) resulted in the consumption, rather than production, of H_2_S by *B. longum* DJO10A (Figure 3(a)). However, increasing the concentration of cystine to 0.16% resulted in a reduction in H_2_S consumption (Figure 4). Additionally, at lower concentrations of H_2_S (0 to 0.1 mM), its production was significantly enhanced when the cysteine concentration was increased to 0.16%. Therefore, the consumption and production of H_2_S are dependent on its initial concentration in the medium, and this process is further regulated by the concentration of cysteine present. These findings suggest that a high concentration of cysteine in the medium is degraded, leading to enhanced H_2_S production by *B. longum* DJO10A.

**Figure 5.**
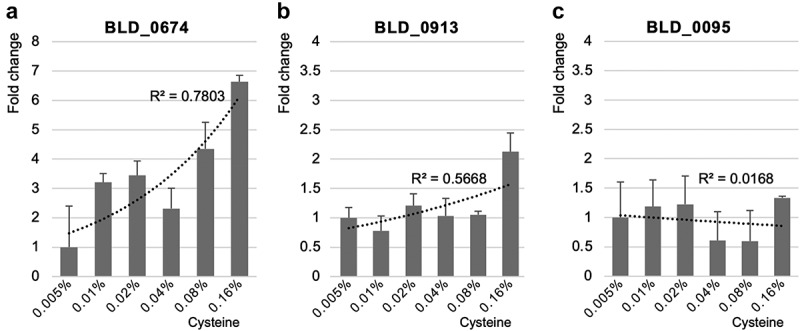
Transcription analysis of cysteine utilizing enzymes from BLD_0674 (a) and BLD_0913 (b), and of non-cysteine utilizing enzyme from BLD_0095 (c), according to the different cysteine concentrations (0.005, 0.01, 0.02, 0.04, 0.08, and 0.16%). The dotted line indicates the trend and the R^2^ value is shown for the correlation between expression levels and cysteine concentration.

**Figure 6.**
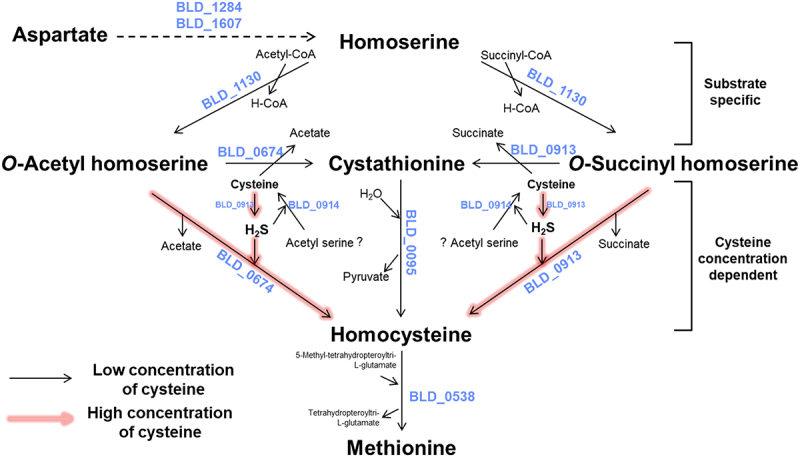
The experimentally confirmed sulfur utilization pathway consisting of trans-sulfurylation pathway at low concentration of cysteine and direct sulfurylation pathway at high concentration of cysteine. These two sub-pathways are regulated by the concentration of cysteine.

To extend our understanding of this, a transcriptional analysis of two H_2_S-utilizing genes (BLD_0674 and BLD_0913) and a specific gene (BLD_0095) converting cystathionine to homocysteine without H_2_S was conducted. Interestingly, the expression patterns of the H_2_S-utilizing genes were proportional to the concentration of cysteine (Figures 5a,b). These genes are associated with the bioconversion of *O*-acetyl/*O*-succinyl homoserine to homocysteine with H_2_S produced from cysteine degradation (Figure 3(c)). Therefore, these genes were up-regulated due to cysteine degradation and H_2_S utilization. However, the expression pattern of the cystathionine-converting gene was consistent given that this gene is not associated with cysteine degradation or H_2_S utilization (Figure 5(c)). This result suggests the possible preference of a specific pathway depending on a high concentration of cysteine. Based on these results, the trans-sulfurylation pathway may be cysteine concentration-independent, given the lack of a response of BLD_0095 to the cysteine concentration, whereas the direct sulfurylation pathway may be cysteine concentration-dependent, as BLD_0674 and BLD_0913 are inducible at high cysteine concentration. Therefore, these results suggest two possible cases without methionine: (i) both the trans- and direct sulfurylation pathways are equally activated at a low cysteine concentration, or (ii) the direct sulfurylation pathway is preferentially more activated than the trans-sulfurylation pathway at a high cysteine concentration (Figure 6).

To identify the metabolic pathway most favorable for methionine biosynthesis, a kinetic study of the specific enzymes was undertaken to determine pathway preference (Table S7). Homoserine transacetylase (BLD_1130) could catalyze with acetyl-CoA (*K*_m_ = 1.318 mM) and succinyl-CoA (*K*_m_ = 2.648 mM) to produce *O*-acetyl homoserine and *O*-succinyl homoserine, respectively. From this result, *B. longum* DJO10A or BLD_1130 may prefer to use acetyl-CoA to produce *O*-acetyl homoserine. A previous transcriptional analysis of BLD_0674 and BLD_0913 demonstrated that the transcription level of BLD_0674 was significantly higher than that of BLD_0913 in the presence of cysteine without methionine (Figure 1(d)). This finding likely reflects the preference for *O*-acetyl homoserine biosynthesis rather than *O*-succinyl homoserine biosynthesis when cysteine is available. In the presence of *O*-acetyl homoserine, BLD_0674 could be involved in the reaction with cysteine (*K*_m_ = 1.234 mM) or H_2_S (*K*_m_ = 3.351 mM), indicating a preference for cysteine. Conversely, in the presence of *O*-succinyl homoserine, BLD_0913 is implicated in the synthesis of cystathionine using cysteine (*K*_m_ = 1.583 mM), the formation of homocysteine using H_2_S (*K*_m_ = 3.948 mM), or the degradation of cysteine to produce H_2_S (*K*_m_ = 0.513 mM). In short, BLD_0913 shows a preference for cysteine in the synthesis of cystathionine and also possesses the ability to degrade cysteine to yield H_2_S. BLD_0095 is exclusively involved in the conversion of cystathionine to homocysteine (*K*_m_ = 2.118 mM). Consequently, *B. longum* DJO10A prefers the specific methionine biosynthesis pathway through homoserine, *O*-acetyl homoserine, homocysteine, and methionine in the presence of cysteine but the absence of methionine (Figure 6).

### Presence and abundance of similar enzymes in other bacterial species and human microbiome

To determine if the enzymes BLD_0674 and BLD_0913 are present in other bacterial species, sequence similarity network (SSN) analysis was performed using the Enzyme Function Initiative-Enzyme similarity Tool (EFI-EST). The results were filtered to analyze the top five clusters with the highest cluster node counts. For BLD_0674, the clusters were primarily composed of *Bifidobacterium* spp., but also included other genera such as *Bacteroides, Parabacteroides, and Faecalibacterium*. Most *Bifidobacterium* spp. were found to belong to cluster 1, with a total node count of 190, indicating their significant presence in this cluster (Figure S2A). In contrast, for BLD_0913, *Bifidobacterium* species were distributed across clusters 1, 2, and 3, with *B. longum* being the most abundant in cluster 1 (52 nodes), and other species including *B. breve* and *B. pseudolongum* in cluster 2 and 3, respectively (Figure S2B). These results provide insight into the prevalence of BLD_0674 and BLD_0913 enzymes among different bacterial species, highlighting their potential roles in microbial physiology. Additionally, the abundance of BLD_0674 and BLD_0913 enzymes was assessed using 380 metagenome samples from the Human Microbiome Project. For BLD_0674, Clusters 1, 2, and 4 were present, with an average abundance of 3.37, which was highest in stool metagenome samples (Figure S2C). Notably, Cluster 2 had the highest abundance at 1.76. Conversely, for BLD_0913, Clusters 1 and 5 were detected in human samples. However, unlike BLD_0674, only Cluster 1 was detected in stool samples, with a very low average abundance of 0.039 (Figure S2D). These findings suggest different distribution patterns and abundances of BLD_0674 and BLD_0913 in the human microbiome.

### *Suggestion of a novel sulfur utilization pathway for* B. longum

*B. longum* synthesizes amino acids from carbohydrate precursors produced via the F6PPK pathway and the partial TCA cycle with glucose.^5^ However, the mechanisms underlying sulfur utilization for the biosynthesis of sulfur-containing amino acids, such as cysteine and methionine, remain unclear. To elucidate this, the complete genome sequence of *B. longum* DJO10A was re-analyzed, leading to the identification of genes associated with sulfur utilization. Their transcription patterns were examined under varying concentrations of cysteine and H_2_S, and their enzyme activities were assessed. Based on these findings, a novel sulfur utilization pathway of *B. longum* was proposed, encompassing both trans- and direct sulfurylation pathways (Figure 6).

Additionally, an enzyme kinetic study revealed that the bioconversion of homoserine to *O*-acetyl homoserine is preferred over the bioconversion to *O*-succinyl homoserine, suggesting that BLD_1130 may prefer acetyl-CoA as a substrate. Furthermore, a subsequent transcription analysis of BLD_0674 and BLD_0913 showed that the gene expression level of BLD_0674 was 3.5-fold higher than that of BLD_0913 with 0.16% cysteine in the medium (Figures 5a,b), suggesting that the direct bioconversion of *O*-acetyl homoserine to homocysteine by BLD_0674 is preferred. Therefore, these results substantiate that homoserine is preferably converted to *O*-acetyl homoserine with acetyl-CoA and then directly converted to homocysteine, which is the main bioconversion pathway from homoserine to homocysteine in *B. longum*. Finally, this homocysteine is converted to methionine as a final product by BLD_0538.

All of these results have been systematically organized, leading to the proposal of a novel sulfur utilization pathway. This pathway includes both trans- and direct sulfurylation pathways, ultimately resulting in the production of methionine (Figure 6). However, this proposed pathway does not produce cysteine but rather utilizes it for H_2_S production, thereby substantiating the cysteine auxotrophy of *B. longum*. Consequently, cysteine is identified as a crucial compound for initiating this pathway for methionine biosynthesis.

## Discussion

Cysteine is a thiol group (−SH)-containing amino acid. This thiol side chain is susceptible to oxidation, forming disulfide bonds that contribute to the tertiary structure of proteins. Additionally, free cysteine can be oxidized to cystine through dimerization with a disulfide bond, aiding in the defense mechanism against oxidative stress in lactic acid bacteria.^36^ Furthermore, while the sulfhydryl group of cysteine is easily oxidized, cysteine can react with oxygen. Due to this oxygen reactivity, cysteine is often used to reduce oxidative stress, promoting the growth of intestinal bacteria under anaerobic conditions.^37^ Therefore, most intestinal bacteria have a cysteine biosynthesis pathway from a sulfate, sulfite, or sulfide.

*E. coli* and *S*. Typhimurium possess assimilatory sulfate reduction pathways that include phosphoadenosine-5’-phosphosulfate (PAPS) reductase to obtain inorganic sulfur from the gut environment. Extracellular sulfate (SO_4_^2-^) is transported into bacterial cells via the *SulP* (sulfate permease), *CysP* (sulfate/thiosulfate transport system substrate-binding protein), and *CysZ* (sulfate transport protein) families.^10^ Intracellular sulfate is adenylated to adenosine 5’-phosphate (APS), which is then reduced to PAPS. Subsequently, PAPS is reduced to sulfite (SO_3_^2-^) by PAPS reductase, and sulfite is further reduced to sulfide (S^2^- or H_2_S) by sulfite reductase. Finally, cysteine is synthesized from *O*-acetylserine and hydrogen sulfide (H_2_S) using *CysK* (cysteine synthase). Cysteine is then used for the synthesis of methionine.^11,12^ Additionally, intestinal sulfate-reducing bacteria such as *Desulfovibrio*, *Desulfobacter*, and *Desulfotomaculum* employ a unique dissimilatory sulfate reduction pathway, which lacks APS kinase and PAPS reductase. In this pathway, APS reductase converts APS directly to sulfite, which is then used for cysteine and methionine synthesis.^13^ Interestingly, only a few genera in gut microbiota possess a sulfite respiration pathway. The intestinal bacterium *Bilophila wadsworthia* degrades taurine to obtain sulfite,^14^ which is reduced to H_2_S by dissimilatory sulfite reductase and used for the biosynthesis of cysteine and methionine.

Furthermore, intestinal bacteria utilize sulfide for cysteine and methionine biosynthesis. Cysteine can be degraded to produce sulfide. Some intestinal pathogens such as *Helicobacter pylori*, *Prevotella*, and *Fusobacterium* use cysteine to produce H_2_S by cysteine desulfhydrase, suggesting that this enzyme may be an important producer of H_2_S in the human colon.^8^ In addition, other intestinal bacteria such as *Pseudomonas aeruginosa*, *Staphylococcus aureus*, and *E. coli* can produce H_2_S from cysteine degradation using cystathionine β-synthase, cystathionine γ-lyase, or 3-mercaptopyruvate sulfurtransferase, respectively.^35^ Interestingly, the intestinal bacterium *Lactobacillus casei* FAM18110 has an *O*-acetylserine sulfhydrylase with dual activities, specifically cysteine desulfurization activity for cysteine degradation and H_2_S production as well as sulfhydrylation activity for cysteine biosynthesis with H_2_S.^31^ This sulfide created by cysteine degradation is used for the biosynthesis of methionine as well as cysteine. Although intestinal sulfide-utilizing bacteria may use the produced H_2_S for the biosynthesis of cysteine and methionine, this biosynthesis pathway is not yet clearly understood. In this study, *B. longum* also degrades cysteine to produce H_2_S, and it is used for the biosynthesis of methionine in the sulfur utilization pathway. However, *B. longum* uses cystathionine β-lyase for cysteine degradation, which is different from other cysteine-degrading enzymes for H_2_S production in other sulfide-utilizing intestinal bacteria.

*B. longum* produces only methionine, not cysteine, due to its cysteine auxotroph. Previously, this auxotrophic feature of bifidobacteria was experimentally validated with bifidobacteria, supporting this.^15^ While *B. bifidum* is also a cysteine auxotroph, the suggested metabolic pathway for sulfur amino acid in *B. bifidum* PRL2010 can be substantiated and updated by the newly suggested mechanism of methionine biosynthesis of *B. longum* DJO10A^15:^ (i) bioconversion of homoserine to *O*-acetyl homoserine, (ii) cysteine degradation for H_2_S production, and (iii) the direct sulfurylation pathway for methionine biosynthesis (direct bioconversion of *O*-acetyl/*O*-succinyl homoserine with H_2_S to homocysteine). Firstly, *B. bifidum* PRL2010 has the BBPR_1654 homologous to BLD_1130 (88.95% protein sequence identity), which converts homoserine with acetyl-CoA to *O*-acetyl homoserine and converts homoserine with succinyl-CoA to *O*-succinyl homoserine, as this enzyme has dual functions depending on the added substrate (Figure 6). However, BBPR_1654 was predicted to have only one function for the bioconversion of homoserine with succinyl-CoA to *O*-succinyl homoserine. The presence of BBPR_1213 in *B. bifidum* PRL2010, homologous to BLD_0674 (95.43% protein sequence identity) converting *O*-acetyl homoserine to homocysteine, supports this missing (homoserine, *O*-acetyl homoserine, homocysteine) pathway in the suggested metabolic pathway for the sulfur amino acid of *B. bifidum* PRL2010. Secondly, BBPR_1343 is homologous to BLD_0913 (cystathionine γ-synthase; 83.07% protein sequence identity) with bioconversion activity of cysteine to H_2_S (Figures 3b,c), suggesting that *B. bifidum* PRL2010 may also have cysteine degradation activity for H_2_S production. Furthermore, a direct sulfurylation pathway is missing in the suggested metabolic pathway for sulfur amino acid, despite the prediction that H_2_S is produced from cysteine degradation by BBPR_1343 and that *O*-acetyl/*O*-succinyl homoserine with H_2_S is directly converted to homocysteine by BBPR_1213 and BBPR_1343 (Figure 6). Therefore, the sulfur utilization pathway of *B. bifidum* PRL2010 needs to be experimentally confirmed for completion of the pathway for methionine biosynthesis. Moreover, it has been reported that introducing the direct sulfurylation pathway from *Cyclobacterium marinum* or *Deinococcus geothermalis* to *E. coli* (which only has the trans-sulfurylation pathway) enhanced methionine biosynthesis in *E. coli*. ^8^ Based on this finding, even though bifidobacteria do not have a sulfate assimilation pathway, methionine biosynthesis in bifidobacteria may be efficient and flexible due to the dual-pathway mechanism.

Therefore, this study reports on the complete sulfur utilization pathway for methionine biosynthesis in bifidobacteria and on the complete direct sulfurylation pathway in sulfide-utilizing intestinal bacteria. Furthermore, while the trans- and direct sulfurylation pathways work together for methionine biosynthesis at low concentrations of cysteine, the direct bioconversion to homocysteine in direct sulfurylation pathway becomes the preferred pathway for methionine biosynthesis at high concentrations of cysteine, indicating that two pathways are regulated by the cysteine concentration in the medium (Figure 5).

This complete sulfur utilization pathway of *B. longum* may be associated with adaptation and survival in the gut environment. Cysteine in the gut environment is generally obtained from food protein digestion and cysteine-producing intestinal bacteria. Therefore, the cysteine biosynthesis activity is most likely lost but the cysteine acquisition and utilization activities of *B. longum* remain for its adaptation and survival in the gut ecosystem, substantiating its cysteine auxotrophic feature. After cysteine acquisition, most of the intestinal bacteria can degrade cysteine to produce H_2_S. However, excessive H_2_S has been reported to promote colonic mucosal inflammation at high concentrations.^33^ Therefore, the utilization of produced H_2_S for methionine biosynthesis by *B. longum* and other intestinal bacteria may be useful for the reduction of toxic H_2_S concentrations in the gut. Consequently, this complete sulfur utilization pathway would provide extensive insight into the essential biosynthesis mechanism of sulfur-containing amino acids, enhancing our understanding of the adaptation and survival of *B. longum* and other intestinal bacteria in the gut environment.

Our study focuses on the sulfur utilization pathways of *Bifidobacterium longum*, with broader implications suggesting significant generalization of the findings. Supplementary Figure S2 reveals the presence of these enzymes in diverse bacterial species. Notably, BLD_0674 in strain DJO10A is shared among bifidobacteria and other gut bacteria such as *Bacteroides*, *Parabacteroides*, and *Faecalibacterium*. This indicates that the direct sulfurylation pathway mediated by BLD_0674 for methionine biosynthesis from *O*-acetylhomoserine is prevalent among gut bacteria. Conversely, BLD_0913 is predominantly present in the *Bifidobacterium* genus, suggesting that the direct sulfurylation pathway by BLD_0913 for methionine biosynthesis from *O*-succinyl homoserine is specific to *Bifidobacterium*. Interestingly, a few other gut bacteria, including *Streptomyces* and *Clavibacter*, also possess BLD_0913 homologs. These bacteria, which belong to the same class as *Bifidobacterium* (Actinomycetia), indicate that the presence of BLD_0913 homologs may represent a distinct methionine biosynthesis pathway differentiated from other gut bacteria. The “dual-pathway mechanism” for methionine biosynthesis likely confers efficiency and flexibility under the harsh conditions of the gut environment, enhancing the survival and proliferation of bifidobacteria. Given that bifidobacteria lack a sulfur assimilation pathway, this dual-pathway mechanism is crucial for their survival. The pathway for obtaining H_2_S through the degradation of cysteine, mediated by BLD_0913 homologs, may have been acquired from evolutionarily related gut bacteria, though this hypothesis remains to be elucidated. These insights underscore the broader ecological and evolutionary significance of sulfur metabolism pathways. Additionally, BLD_0674 shows a higher abundance in stool samples compared to BLD_0913, underscoring a preference for *O-*acetyl homoserine over *O-*succinyl homoserine. This preference is consistent with our kinetic analyses indicating a higher affinity for *O-*acetyl homoserine. Furthermore, previous studies have shown that most gut microbiota also prefers *O-*acetylhomoserine,^7,38^ reinforcing the importance of this substrate in gut microbial sulfur metabolism.

## Supplementary Material

Supplementary Data_Gut microbes_revision.docx

## References

[1] Christl SU, Gibson GR, Cummings JH. Role of dietary sulphate in the regulation of methanogenesis in the human large intestine. Gut. 1992;33(9):1234–19. doi:10.1136/gut.33.9.1234. 1427377 PMC1379493

[2] van Hylckama Vlieg JE, Veiga P, Zhang C, Derrien M, Zhao L. Impact of microbial transformation of food on health—from fermented foods to fermentation in the gastro-intestinal tract. Curr Opin Biotechnol. 2011;22(2):211–219. doi:10.1016/j.copbio.2010.12.004. 21247750

[3] Flint HJ, Scott KP, Duncan SH, Louis P, Forano E. Microbial degradation of complex carbohydrates in the gut. Gut Microbes. 2012;3(4):289–306. doi:10.4161/gmic.19897. 22572875 PMC3463488

[4] Turnbaugh PJ, Ley RE, Mahowald MA, Magrini V, Mardis ER, Gordon JI. An obesity-associated gut microbiome with increased capacity for energy harvest. Nature. 2006;444(7122):1027–1031. doi:10.1038/nature05414. 17183312

[5] Lee J-H, O’Sullivan DJ. Genomic insights into bifidobacteria. Microbiol Mol Biol Rev. 2010;74(3):378–416. doi:10.1128/MMBR.00004-10. 20805404 PMC2937518

[6] Sela DA, Garrido D, Lerno L, Wu S, Tan K, Eom H-J, Joachimiak A, Lebrilla CB, Mills DA. *Bifidobacterium longum* subsp. *infantis* ATCC 15697 α-fucosidases are active on fucosylated human milk oligosaccharides. Appl Environ Microbiol. 2012;78(3):795–803. doi:10.1128/AEM.06762-11. 22138995 PMC3264123

[7] Brewster JL, Pachl P, McKellar JLO, Selmer M, Squire CJ, Patrick WM. Structures and kinetics of *Thermotoga maritima* MetY reveal new insights into the predominant sulfurylation enzyme of bacterial methionine biosynthesis. J Biol Chem. 2021;296:100797. doi:10.1016/j.jbc.2021.100797. 34019879 PMC8191291

[8] Gruzdev N, Hacham Y, Haviv H, Stern I, Gabay M, Bloch I, Amir R, Gal M, Yadid I. Conversion of methionine biosynthesis in *Escherichia coli* from trans- to direct- sulfurylation enhances extracellular methionine levels. Microb Cell Fact. 2023;22(1):151. doi:10.1186/s12934-023-02150-x. 37568230 PMC10416483

[9] Bandyopadhyay P, Pramanick I, Biswas R, Sabarinath PC, Sreedharan S, Singh S, Rajmani RS, Laxman S, Dutta S, Singh A, et al. S -Adenosylmethionine–responsive cystathionine β-synthase modulates sulfur metabolism and redox balance in *Mycobacterium tuberculosis*. Sci Adv. 2022;8(25):eabo0097. doi:10.1126/sciadv.abo0097. 35749503 PMC9232105

[10] Schmidt A, Jäger K. Open questions about sulfur metabolism in plants. Annu Rev Plant Physiol Plant Mol Biol. 1992;43(1):325–349. doi:10.1146/annurev.pp.43.060192.001545.

[11] Hullo M-F, Auger S, Soutourina O, Barzu O, Yvon M, Danchin A, Martin-Verstraete I. Conversion of methionine to cysteine in *Bacillus subtilis* and its regulation. J Bacteriol. 2007;189(1):187–197. doi:10.1128/JB.01273-06. 17056751 PMC1797209

[12] Yeom H-J, Hwang B-J, Lee M-S, Kim Y-H, Lee H-S. Regulation of enzymes involved in methionine biosynthesis in *Corynebacterium glutamicum*. J Microbiol Biotechnol. 2004;14:373–378.

[13] Kopriva S, Fritzemeier K, Wiedemann G, Reski R. The putative moss 3′-phosphoadenosine-5′-phosphosulfate reductase is a novel form of adenosine-5′-phosphosulfate reductase without an iron-sulfur cluster. J Biol Chem. 2007;282(31):22930–22938. doi:10.1074/jbc.M702522200. 17519237

[14] Laue H, Denger K, Cook AM. Taurine reduction in anaerobic respiration of *Bilophila wadsworthia* RZATAU. Appl Environ Microbiol. 1997;63(5):2016–2021. doi:10.1128/aem.63.5.2016-2021.1997. 9143131 PMC168491

[15] Ferrario C, Duranti S, Milani C, Mancabelli L, Lugli GA, Turroni F, Mangifesta M, Viappiani A, Ossiprandi MC, van Sinderen D, et al. Exploring amino acid auxotrophy in *Bifidobacterium bifidum* PRL2010. Front Microbiol. 2015;6:1331. doi:10.3389/fmicb.2015.01331. 26635786 PMC4656816

[16] Carbonero F, Benefiel AC, Alizadeh-Ghamsari AH, Gaskins HR. Microbial pathways in colonic sulfur metabolism and links with health and disease. Front Physiol. 2012;3:448. doi:10.3389/fphys.2012.00448. 23226130 PMC3508456

[17] Schäffer AA, Aravind L, Madden TL, Shavirin S, Spouge JL, Wolf YI, Koonin EV, Altschul SF. Improving the accuracy of PSI-BLAST protein database searches with composition-based statistics and other refinements. Nucleic Acids Res. 2001;29(14):2994–3005. doi:10.1093/nar/29.14.2994. 11452024 PMC55814

[18] Jones P, Binns D, Chang HY, Fraser M, Li W, McAnulla C, McWilliam H, Maslen J, Mitchell A, Nuka G, et al. InterProScan 5: genome-scale protein function classification. Bioinformatics. 2014;30(9):1236–1240. doi:10.1093/bioinformatics/btu031. 24451626 PMC3998142

[19] Moriya Y, Itoh M, Okuda S, Yoshizawa AC, Kanehisa M. KAAS: an automatic genome annotation and pathway reconstruction server. Nucleic Acids Res. 2007;35(Web Server):W182–5. doi:10.1093/nar/gkm321. 17526522 PMC1933193

[20] Seemann T. Prokka: rapid prokaryotic genome annotation. Bioinformatics. 2014;30(14):2068–2069. doi:10.1093/bioinformatics/btu153. 24642063

[21] Page AJ, Cummins CA, Hunt M, Wong VK, Reuter S, Holden MT, Fookes M, Falush D, Keane JA, Parkhill J, et al. Roary: rapid large-scale prokaryote pan genome analysis. Bioinformatics. 2015;31(22):3691–3693. doi:10.1093/bioinformatics/btv421. 26198102 PMC4817141

[22] Richter M, Rosselló-Móra R. Shifting the genomic gold standard for the prokaryotic species definition. Proc Natl Acad Sci USA. 2009;106(45):19126–19131. doi:10.1073/pnas.0906412106. 19855009 PMC2776425

[23] Livak KJ, Schmittgen TD. Analysis of relative gene expression data using real-time quantitative PCR and the 2^− ΔΔC^_T_ method. Methods. 2001;25(4):402–408. doi:10.1006/meth.2001.1262. 11846609

[24] Sambrook J, Fritsche E, Maniatis T. Molecular cloning: a laboratory manual. 2nd ed. NewYork, USA: Cold Spring Harbor, Cold Harbor Laboratory Press; 1989.

[25] Rocchiccioli M, Moschini R, Cappiello L, Balestri F, Cappiello M, Mura U, Del-Corso A. Colorimetric coupled enzyme assay for cystathionine β-synthase. Anal Sci. 2016;32(8):901–906. doi:10.2116/analsci.32.901. 27506718

[26] Siegel LM. A direct microdetermination for sulfide. Anal Biochem. 1965;11(1):126–132. doi:10.1016/0003-2697(65)90051-5. 14328633

[27] Hanes CS. Studies on plant amylases: the effect of starch concentration upon the velocity of hydrolysis by the amylase of germinated barley. Biochem J. 1932;26:1406–1421. 16744959 10.1042/bj0261406 PMC1261052

[28] Zallot R, Oberg N, Gerlt JA. The EFI web resource for genomic enzymology tools: leveraging protein, genome, and metagenome databases to discover novel enzymes and metabolic pathways. Biochemistry. 2019;58(41):4169–4182. doi:10.1021/acs.biochem.9b00735. 31553576 PMC7057060

[29] Oberg N, Zallot R, Gerlt JA. EFI-EST, EFI-GNT, and EFI-CGFP: enzyme function initiative (EFI) web resource for genomic enzymology tools. 2023. J Mol Biol. 2023;435(14):168018. doi:10.1016/j.jmb.2023.168018. 37356897 PMC10291204

[30] Lee J-H, Karamychev V, Kozyavkin S, Mills D, Pavlov A, Pavlova N, Polouchine NN, Richardson PM, Shakhova VV, Slesarev AI, et al. Comparative genomic analysis of the gut bacterium *Bifidobacterium longum* reveals loci susceptible to deletion during pure culture growth. BMC Genomics. 2008;9(1):247. doi:10.1186/1471-2164-9-247. 18505588 PMC2430713

[31] Bogicevic B, Berthoud H, Portmann R, Meile L, Irmler S. CysK from *Lactobacillus casei* encodes a protein with O-acetylserine sulfhydrylase and cysteine desulfurization activity. Appl Microbiol Biotechnol. 2012;94(5):1209–1220. doi:10.1007/s00253-011-3677-5. 22113557

[32] Irmler S, Schäfer H, Beisert B, Rauhut D, Berthoud H. Identification and characterization of a strain-dependent cystathionine β/γ-lyase in *Lactobacillus casei* potentially involved in cysteine biosynthesis. FEMS Microbiol Lett. 2009;295(1):67–76. doi:10.1111/j.1574-6968.2009.01580.x. 19473252

[33] Wüthrich D, Wenzel C, Bavan T, Bruggmann R, Berthoud H, Irmler S. Transcriptional regulation of cysteine and methionine metabolism in *Lactobacillus paracasei* FAM18149. Front Microbiol. 2018;9:1261. doi:10.3389/fmicb.2018.01261. 29942297 PMC6004538

[34] Hwang BJ, Yeom HJ, Kim Y, Lee HS. *Corynebacterium glutamicum* utilizes both transsulfuration and direct sulfhydrylation pathways for methionine biosynthesis. J Bacteriol. 2002;184(5):1277–1286. doi:10.1128/JB.184.5.1277-1286.2002. 11844756 PMC134843

[35] Shatalin K, Shatalina E, Mironov A, Nudler E. H_2_S: a universal defense against antibiotics in bacteria. Science. 2011;334(6058):986–990. doi:10.1126/science.1209855. 22096201

[36] Turner MS, Woodberry T, Hafner LM, Giffard PM. The *bspA* locus of *Lactobacillus fermentum* BR11 encodes an L-cystine uptake system. J Bacteriol. 1999;181(7):2192–2198. doi:10.1128/JB.181.7.2192-2198.1999. 10094698 PMC93633

[37] Carlsson J, Granberg G, Nyberg G, Edlund M. Bactericidal effect of cysteine exposed to atmospheric oxygen. Appl Environ Microbiol. 1979;37(3):383–390. doi:10.1128/aem.37.3.383-390.1979. 453819 PMC243226

[38] Ferla MP, Patrick WM. Bacterial methionine biosynthesis. Microbiology. 2014;160(8):1571–1584. doi:10.1099/mic.0.077826-0. 24939187

