# Supplementary material for: Regulatory mechanism of cysteine-dependent methionine biosynthesis in Bifidobacterium longum: insights into sulfur metabolism in gut microbiota
Source: Gut Microbes. 2024 Oct 28;16(1):2419565. doi: 10.1080/19490976.2024.2419565 (PMC11540073; doi:10.1080/19490976.2024.2419565)
Supplement: Supplementary Data_Gut microbes_revision.docx [file KGMI_A_2419565_SM9544.docx]

**Supplementary Information for**

**Regulatory Mechanism of Cysteine-dependent Methionine Biosynthesis in *Bifidobacterium longum*: Insights into Sulfur Metabolism in Gut Microbiota**

**You-Tae Kim^a,b,c,†^, Joon-Gi Kwon^a,b,c^, Daniel J. O’Sullivan^d^, and Ju-Hoon Lee^a,b,c,*^**

^a^Department of Agricultural Biotechnology, Seoul National University, Seoul 08826, Republic of Korea

^b^Department of Food and Animal Biotechnology, Seoul National University, Seoul 08826, Republic of Korea

^c^Center for Food and Bioconvergence, Seoul National University, Seoul 08826, Republic of Korea

^d^Department of Food Science and Nutrition, Center for Microbial and Plant Genomics, University of Minnesota, St. Paul, Minnesota 55108, USA

**^1^To whom correspondence may be addressed:** Dr. Ju-Hoon Lee.

E-mail: [juhlee@snu.ac.kr](mailto:juhlee@snu.ac.kr)

**This PDF file includes:**

Supplementary Figure S1, Table S1, S2, S3, S4, S5, S6, and S7

**Supplementary Figure legend**

**Figure S1. SDS-PAGE gel image of heterologously expressed and purified enzymes.** (A) Expressed enzymes, BLD_1130 (homoserine *O*-acetyltransferase, lane 1), BLD_0913 (cystathionine γ-synthase, lane 2), BLD_0914 (cystathionine β-synthase, lane 3), BLD_0095 (cystathionine β-lyase, lane 4), and BLD_0674 (*O*-acetylhomoserine sulfhydrylase, lane 5), were purified by Ni-NTA column and showed on SDS-PAGE with protein marker (Lane M, BioRad, Hercules, CA). (B) The table lists the locus tags, the number of amino acids, and protein IDs in GenBank.

**Figure S2. Protein sequence similarity network (SSN) and abundance analysis of BLD_0674 and BLD_0913.** (A) protein sequence similarity network for BLD_0674, and (B) BLD_0913, (C) prevalence of BLD_0674 in 380 microbiome samples from healthy human participants, and (D) BLD_0913

**Supplementary Figure S1.**

**
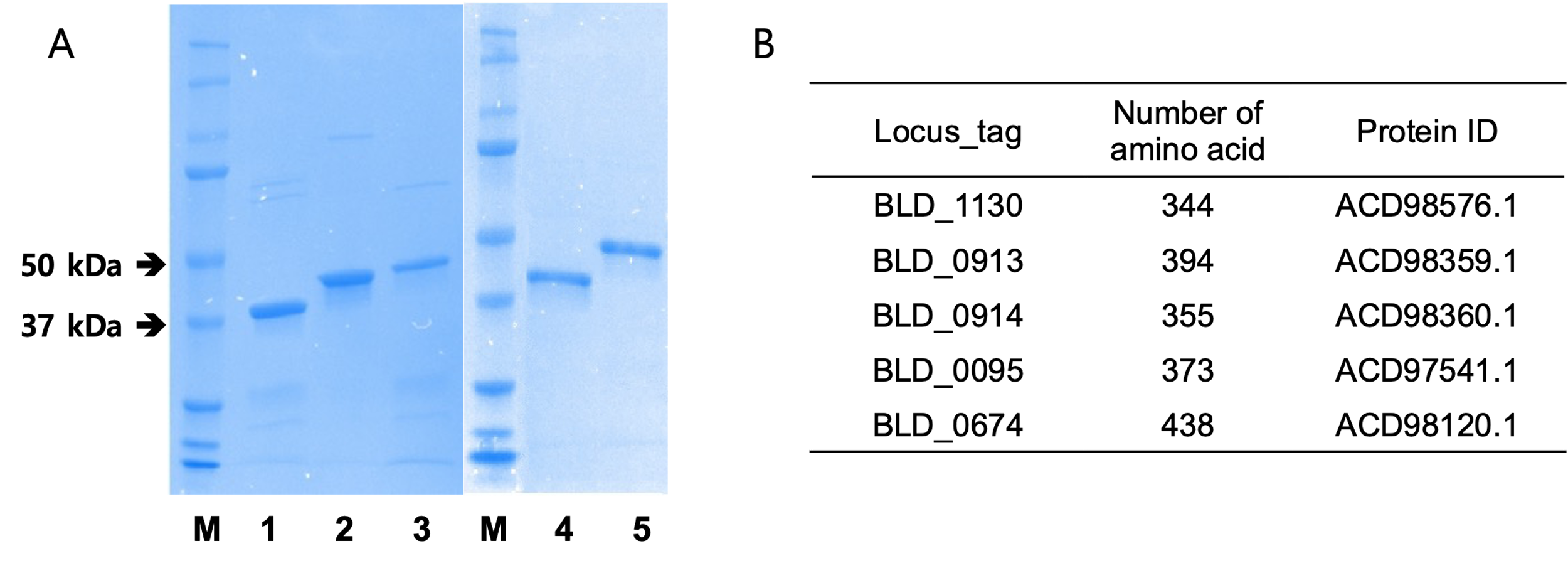
**

**Supplementary Figure S2.**


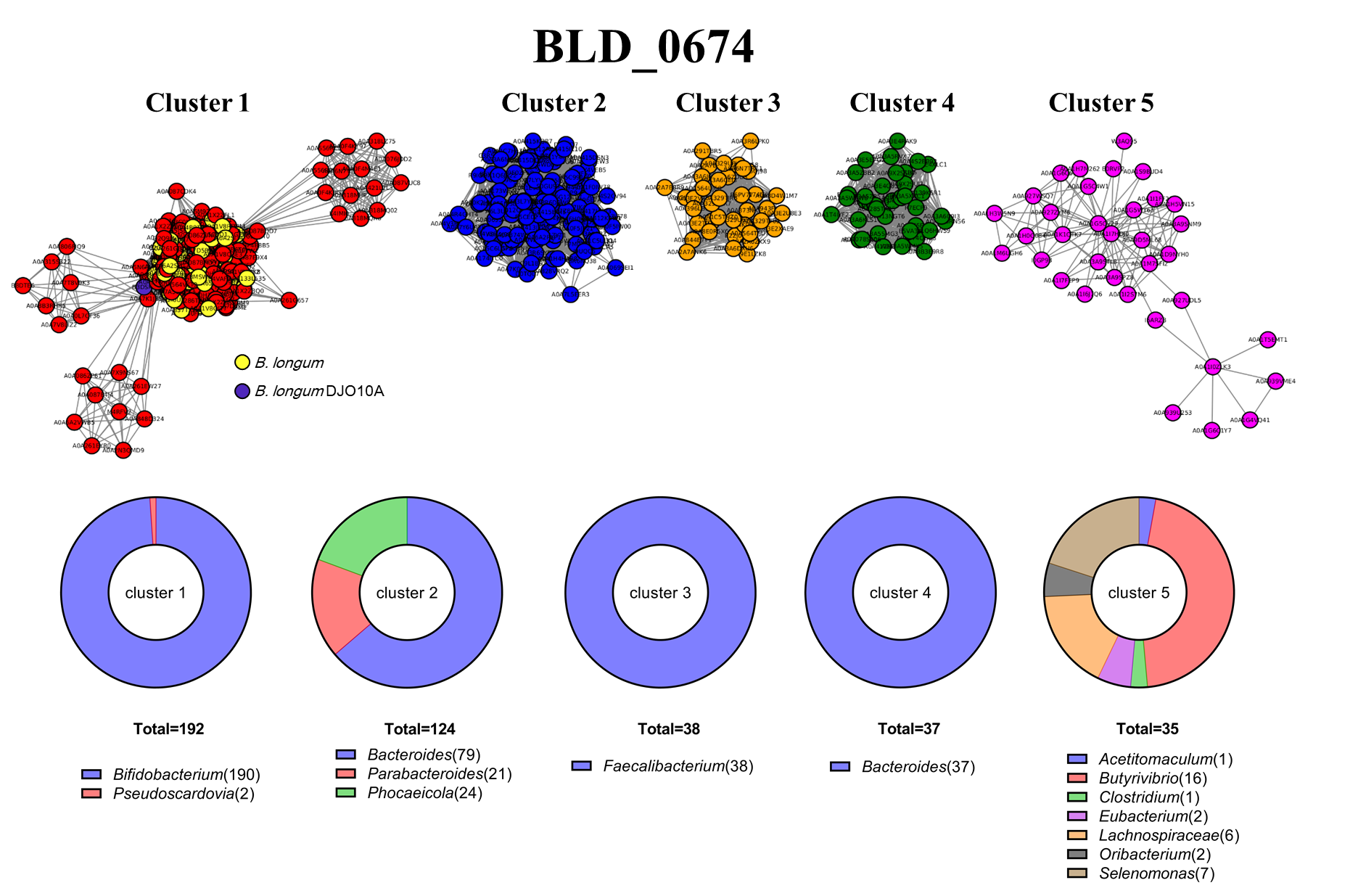
**A**

**
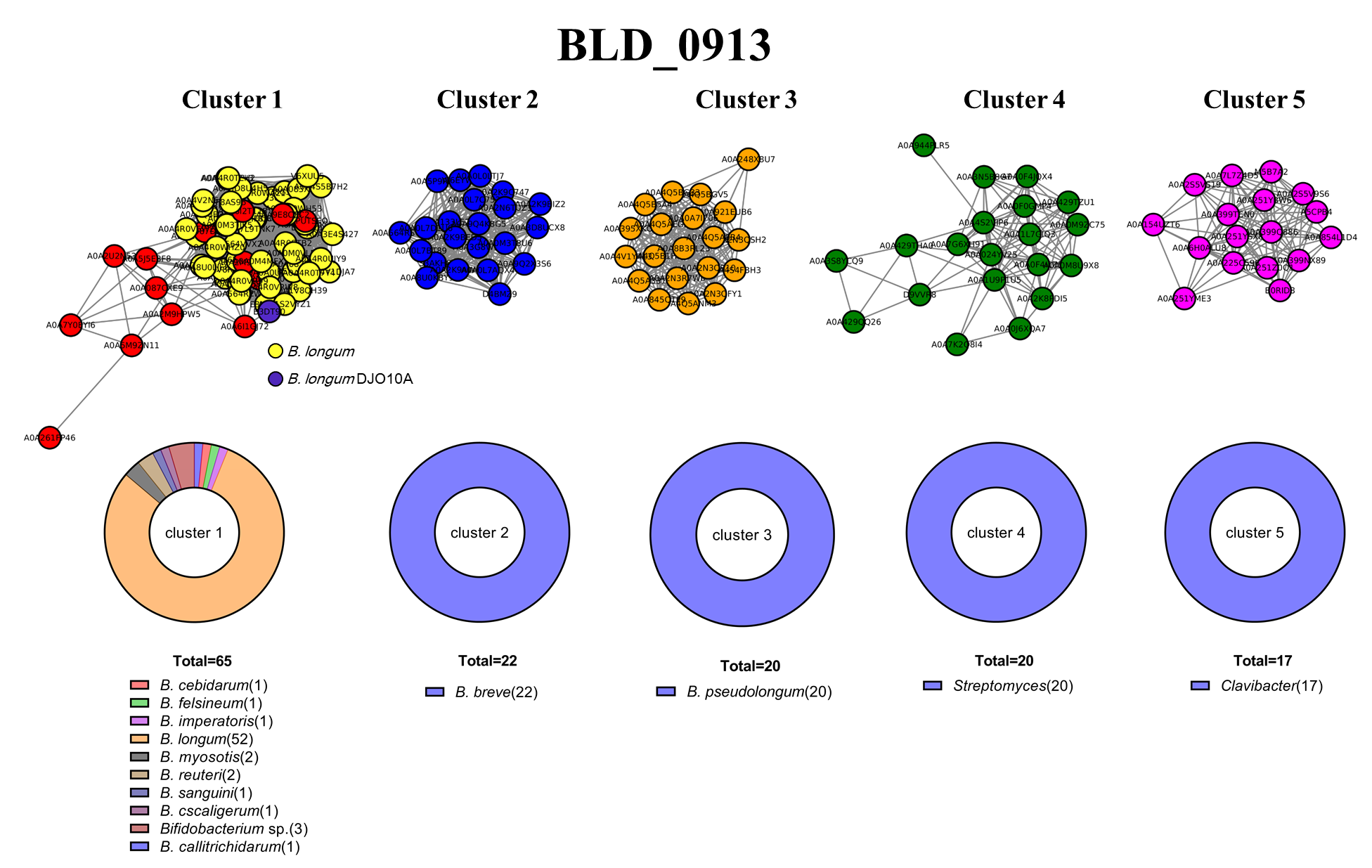
B**

**
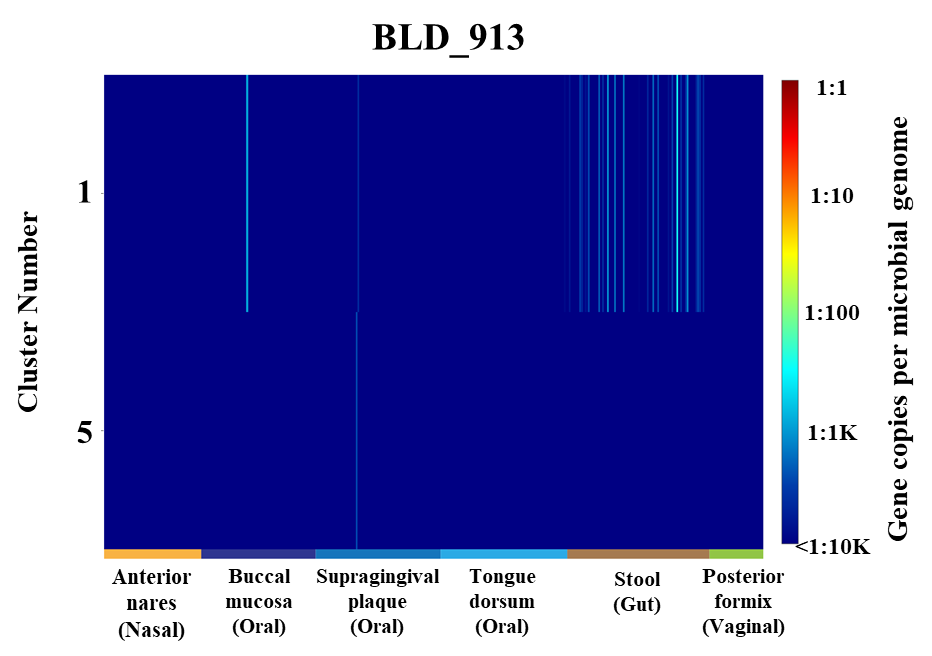

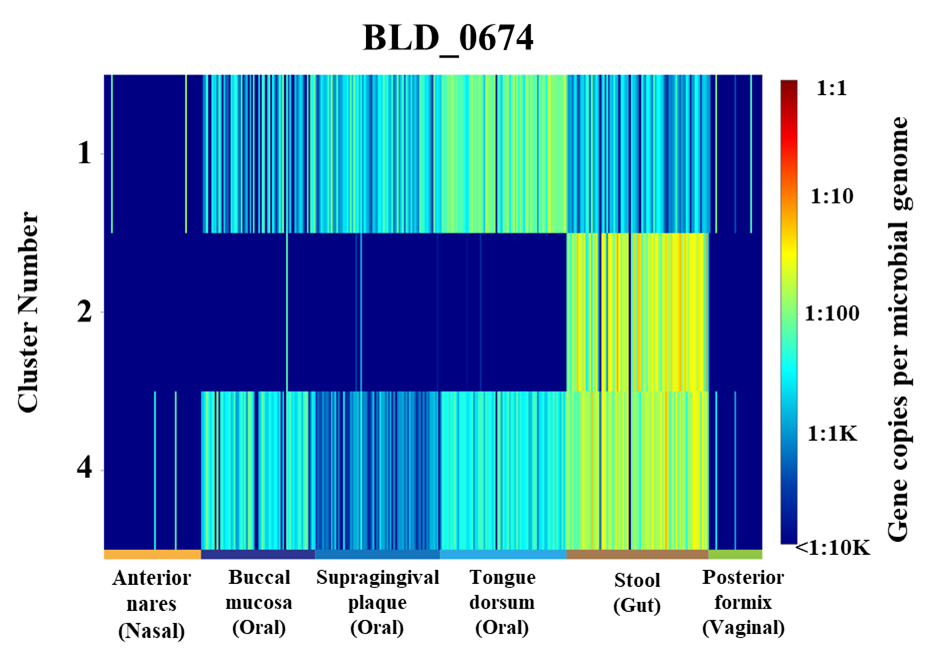
C** **D**

**Table S1. 20 complete genome sequences used for comparative analysis of sulfur utilization pathways**

| **Strain** | **GenBank**  **Accession No.** | **Source** |
| --- | --- | --- |
| *B. longum* subsp. *longum* DJO10A | CP000605.1 | Human |
| *B. adolescentis* DSM 20083 | AP009256.1 | Human |
| *B. bifidum* DSM 20456 | AP012323.1 | Human |
| *B. breve* DSM 20213 | AP012324.1 | Human |
| *B. catenulatum* subsp. *kashiwanohense* DSM 21854 | CP007456.1 | Human |
| *B. longum* subsp. *infantis* DSM 20088 | AP010889.1 | Human |
| *B. scardovii* DSM 13734 | AP012331.1 | Human |
| *B. thermophilum* RBL67 | CP004346.1 | Human |
| *B. pseudocatenulatum* DSM 20438 | AP012330.1 | Human |
| *B. catenulatum* DSM 16992 | AP012325.1 | Human |
| *B. angulatum* DSM 20098 | AP012322.1 | Human |
| *B. dentium* DSM 20436 | AP012326.1 | Human |
| *B. animalis* subsp. *lactis* DSM 10140 | CP003941.1 | Sewage |
| *B. pseudolongum* subsp. *globosum* DSM 20092 | CP017695.1 | Sheep |
| *B. animalis* subsp. *animalis* DSM 20104 | CP002567.1 | Rat |
| *B. choerinum* FMB-1 | CP018044.1 | Cow |
| *B. gallinarum* DSM 20670 | CP035464.1 | Dog |
| *B. asteroides* DSM 20089 | CP017696.1 | Honeybee |
| *B. coryneforme* DSM 20216 | CP007287.1 | Honeybee |
| *B. indicum* DSM 20214 | CP006018.1 | Honeybee |

**Table S2. Bacterial strains and plasmids used in this study**

| Strain or plasmid | Genotype or description | Source or reference |
| --- | --- | --- |
| *B. longum* |  |  |
| DJO10A | Isolated from a healthy young adult’s feces | ([23](#_ENREF_23)). |
| *Bac. subtilis* |  |  |
| ATCC 23857 | Positive control for CDM | ATCC^a^ |
| *E. coli* |  |  |
| DH5α | F^-^ Phi80d*lacZ* ΔM15 Δ(*lacZYA-argF*)U169 *deoR recA1 endA1 hsdR17*(rK-mK+) *phoA supE44 lambda- thi-1*; Cloning host | Novagen |
| BL21(DE3) | F^-^ *ompT* *hsdSB* (r^-^_B_m^-^_B_) *gal dcm* (DE3); Expression host | Novagen |
| Plasmid |  |  |
| pET15b | 5,708-bp, pBR322, N-terminal His_6_ tag, T7 promoter, Amp^r^, Expression vector | Novagen |
| pET21a | 5,443-bp, pBR322, C-terminal His_6_ tag, T7 promoter, Amp^r^, Expression vector | Novagen |
| pET21a-BLD_1130 | 1,032-bp carrying the ORF BLD_1130 without stop codon cloned into *Nhe*I-*Xho*I sites of pET21a | This study |
| pET15b-BLD_0095 | 1,119-bp carrying the ORF BLD_0095 without stop codon cloned into *Nde*I-*Bam*HI sites of pET15b | This study |
| pET15b-BLD_0913 | 1,182-bp carrying the ORF BLD_0913 without stop codon cloned into *Nde*I-*Bam*HI sites of pET15b | This study |
| pET15b-BLD_0914 | 1,065-bp carrying the ORF BLD_0914 without stop codon cloned into *Nde*I-*Bam*HI sites of pET15b | This study |
| pET15b-BLD_0674 | 1,314-bp carrying the ORF BLD_0674 without stop codon cloned into *Nde*I-*Bam*HI sites of pET15b | This study |

^a^, American Type Culture Collection

**Table S3. The composition of chemically defined medium (CDM)**

| Ingredient^a^ | Per liter |
| --- | --- |
| M9 salt | 11.28 g |
| Glucose | 20 g |
| Lactose | 10 g |
| MgCl_2_·6H_2_O  (MgSO_4_·7H_2_O)^b^ | 0.4 g  (0.5 g) |
| CaCl_2_·2H_2_O | 14.7 mg |
| MnCl_2_·4H_2_O | 10 mg |
| FeCl_3_·6H_2_O | 0.027 mg |
| Amino acid mixture^c^ | 1.85 g |
| L-methionine | 0.2 g |
| L-cysteine·HCl | 0.4 g |
| Vitamin solution^d,e^ | 25 ml |

^a^, All reagents were purchased from Sigma-Aldrich.

^b^, MgSO_4_·7H_2_O was used for the condition to give SO_4_^2-^.

^c^, Amino acid mixture: Synthetic complete drop-out: cysteine^-^ and methionine^-^ (Formedium, UK)

^d^, Vitamin solution :10 mg Thiamine, 2 mg Nicotinamide, 2 mg Folic acid, 2 mg Choline chloride, 0.2 mg Riboflavin, and 0.2 mg Biotin in 100 ml of distilled water

^e^, Vitamins solution was added after cooling down of autoclaved medium

**Table S4. Sequences of primers and probes for RT-PCR**

| **Name** | **Type** | **Target genes** | **Sequence (5’ to 3’)** | **Amplicon size (bp)** |
| --- | --- | --- | --- | --- |
| RecA_L2 | Forward primer | *recA*  (BLD_0284) | CTGGCGCAGGTCGAGAAGAG | 203 |
| RecA_R2 | Reverse primer |  | GCCACCACATGCAATGCGAG |  |
| RecA_P2 | Probe |  | 6-FMA-AATGCGACTGGGCGACCAGC-BHQ1 |  |
| 0095_L1 | Forward primer | *metC*  (BLD_0095) | GGTGAGGATCTGGCGAACCG | 122 |
| 0095_R1 | Reverse primer |  | TGACGATCCATGCGAAGGGC |  |
| 0095_P1 | Probe |  | 6-FMA-GGCTCCTGCTGAGTGCGACG-BHQ1 |  |
| 0913_L2 | Forward primer | *metB*  (BLD_0913) | TGGCCGAATGGCTCGAATCC | 179 |
| 0913_R2 | Reverse primer |  | CGTGTGGTCCACGAAGTGCT |  |
| 0913_P2 | Probe |  | 6-FMA-GAAATCGCCGCCCGCCAGAT-BHQ1 |  |
| 0914_L1 | Forward primer | *cysK*  (BLD_0914) | TGGCCAATACGATTCCCGGC | 216 |
| 0914_R1 | Reverse primer |  | CGGAGCCGATGACCTTGACC |  |
| 0914_P1 | Probe |  | 6-FMA-AAGGAAGCCTCCAACGGCGC-BHQ1 |  |
| 0674_L3 | Forward primer | *cysD*  (BLD_0674) | ATCACCCAGTCCGGTGACCA | 148 |
| 0674_R3 | Reverse primer |  | CCTGGATGGCGTCCTCGAAC |  |
| 0674_P3 | Probe |  | 6-FMA-GTTTCCGCCGAGAACCCGCA-BHQ1 |  |
| 1130_L4 | Forward primer | *metA*  (BLD_1130) | TCTCCGAAGTGTTCGCGCTC | 185 |
| 1130_R4 | Reverse primer |  | GTTGCGCCACAGCAGATTGG |  |
| 1130_P4 | Probe |  | 6-FMA-ACGGCAAGTACACGCTCGCC-BHQ1 |  |
| 0538_L4 | Forward primer | *metE*  (BLD_0538) | GTCAAGCCCCCGATCGTCTG | 165 |
| 0538_R4 | Reverse primer |  | CTCGTGGGTGATGTCCTCGC |  |
| 0538_P4 | Probe |  | 6-FMA-CGCGCCAACCCGATTACCGT-BHQ1 |  |
| 1130_NheI_F | Forward primer | *metA*  (BLD_1130) | ctaGCTAGCATGCCTATCAAGATCCCCAGTG | 1,035 |
| 1130_XhoI_R | Reverse primer |  | ccgCTCGAGGCCCTGAATCACCCCATACC |  |
| 0095_NdeI_F | Forward primer | *metC*  (BLD_0095) | ggaattcCATATGCCCCGTTACGACTATGC | 1,128 |
| 0095_BamHI_R | Reverse primer |  | cgcGGATCCGATCAGGCAAACACCCCCC |  |
| 0913_NdeI_F | Forward primer | *metB*  (BLD_0913) | ggaattcCATATGTCCGCTGAATACAACGCC | 1,191 |
| 0913_BamHI_R | Reverse primer |  | cgcGGATCCGATACGATCCAACGCCTGCT |  |
| 0914_NdeI_F | Forward primer | *cycK*  (BLD_0914) | ggaattcCATATGACCATCCACAACAGCCTC | 1,074 |
| 0914_BamHI_R | Reverse primer |  | cgcGGATCCGTTATGGAGTCCGTCCCCAG |  |
| 0674_NdeI_F | Forward primer | *cysD*  (BLD_0674) | ggaattcCATATGGCCGAGAACAACAAGAAGTA | 1,323 |
| 0674_BamHI_R | Reverse primer |  | cgcGGATCCCTTGGCCAAGCCGGAAG |  |

*Underlined sequences indicated the locations of the restriction sites.

**Table S5. Re-annotation of sulfur utilization pathway related genes**

| **Locus tag number** | **Original annotation** | **Re-annotation** | **KO** | **BLASTP match** | **Identity**  **Coverage** |
| --- | --- | --- | --- | --- | --- |
| BLD_1130 | Homoserine trans-succinylase | Homoserine *O*-acetyltransferase | K00651 | homoserine O-succinyltransferase [*Bifidobacteriaceae* bacterium MCC01976; GDZ16781.1] | 100%  344/344 |
| BLD_0674 | *O*-acetylhomoserine sulfhydrylase | *O*-acetylhomoserine sulfhydrylase | K01740 | O-acetylhomoserine (thiol)-lyase [*Bifidobacteriaceae* bacterium MCC01972; GDY93546.1] | 100%  438/438 |
| BLD_0101 | *O*-acetylhomoserine sulfhydrylase | Partial sequence of PLP dependent protein | no KO assigned | PLP-dependent transferase [*Bifidobacterium longum*; WP_195449350.1] | 99.23%  129/431 |
| BLD_0100 | Cystathionine β-lyases / cystathionine γ-synthase | Partial sequence of PLP dependent protein | no KO assigned | PLP-dependent transferase [*Bifidobacterium longum*; WP_195449350.1] | 96.77%  60/431 |
| BLD_0095 | Cystathionine β-lyases / cystathionine γ-synthase | Cystathionine β-lyases | K01760 | cystathionine beta-lyase [*Bifidobacterium longum* BIOML-A162; KAB7142714.1] | 98.93%  371/412 |
| BLD_0913 | Cystathionine β-lyases / cystathionine γ-synthase | Cystathionine γ-synthase | K01739 | cystathionine gamma-synthase [*Bifidobacterium* sp.; MBP9487662.1] | 100%  394/394 |
| BLD_1678 | Cystathionine β-lyases / cystathionine γ-synthase | Partial sequence of cystathionine γ-lyase | no KO assigned | Possible cystathionine gamma lyase [*Bifidobacterium longum* subsp. *longum* BBMN68; ADQ02628.1] | 97.96%  49/410 |
| BLD_1679 | Cystathionine β-lyases / cystathionine γ-synthase | Partial sequence of cystathionine γ-lyase | no KO assigned | Possible cystathionine gamma lyase [*Bifidobacterium longum* subsp. *longum* BBMN68; ADQ02628.1] | 91.25%  130/410 |
| BLD_0914 | Cysteine synthase | Cystathionine β-synthase | K01697 | cystathionine beta-synthase [*Bifidobacterium longum* NCC2705; AAN24961.1] | 100%  355/355 |
| BLD_0538 | Cobalamin-independent methionine synthase II | Methionine synthase | K02968 | 5-methyltetrahydropteroyltriglutamate--homocysteine methyltransferase [*Bifidobacteriaceae* bacterium MCC01976; GDZ17220.1] | 100%  767/767 |

**Table S6. Substrates and products from enzyme reaction by LC-MS/MS analysis**

| **Reaction enzyme** | **Substrates** | **Homoserine (119)**^a^ | **Cysteine  (121)** | **Homocysteine  (135)** | **O-acetyl serine  (147)** | **Methionine  (149)** | **O-acetyl  homoserine  (161)** | **O-succinyl  homoserine (219)** | **Cystathionine  (222)** | **LC-MS/MS result** |
| --- | --- | --- | --- | --- | --- | --- | --- | --- | --- | --- |
| BLD_1130 | Acetyl CoA + Homoserine | ++^b^ | - | - | - | - | **++**^c^ | - | - | Figure 2A |
| BLD_1130 | Succinyl CoA + Homoserine | ++ | - | - | - | - | - | **+++** | - | Figure 2B |
| BLD_0674 | O-acetyl homoserine + Cysteine | - | ++ | - | - | - | +++ | - | **+** | Figure 2C |
| BLD_0674 | O-acetyl homoserine + H_2_S | - | - | **+** | - | - | +++ | - | - | Figure 2D |
| BLD_0913 | O-succinyl homoserine + Cysteine | - | ++ | - | - | - | - | ++ | **+** | Figure 2E |
| BLD_0913 | O-succinyl homoserine + H_2_S | - | - | **+** | - | - | - | ++ | - | Figure 2F |
| BLD_0095 | Cystathionine + H_2_O | - | - | **++** | - | - | - | - | ++ | Figure 2G |
| BLD_0914 | O-acetyl serine + H_2_S | - | **+** | - | ++ | - | - | - | - | Figure 2H |

^a^Moleculare weight (m/z)

^b^Intensity of LC-MS/MS was classified as follows: -, not detected (intensity < 250); +, intensity > 50000; ++, intensity >100000; +++, intensity > 150000

^c^Bold indicates the major product from each enzymatic reaction

**Table S7. Kinetic parameters of enzymes involved in sulfur metabolism in *B. longum* DJO10A**

| **Enzyme** | **Substrate** | ***K*_m_** |
| --- | --- | --- |
| BLD_1130 | Acetyl-CoA | 1.318 mM |
|  | Succinyl-CoA | 2.648 mM |
| BLD_0674 | Cysteine (10 mM OAH^a^) | 1.234 mM |
|  | H_2_S (10 mM OAH) | 3.351 mM |
| BLD_0913 | Cysteine (10 mM OSH^b^) | 1.583 mM |
|  | H_2_S (10 mM OSH) | 3.948 mM |
|  | Cysteine (only) | 0.513 mM |
| BLD_0095 | Cystathionine | 2.118 mM |

^a^ OAH, O-acetyl homoserine; the OAH concentration was fixed in 10 mM

^b^ OSH, O-acetyl succinyl homoserine; the OSH concentration was fixed in 10 mM
